# Land use in mountain grasslands alters drought response and recovery of carbon allocation and plant‐microbial interactions

**DOI:** 10.1111/1365-2745.12910

**Published:** 2017-12-20

**Authors:** Stefan Karlowsky, Angela Augusti, Johannes Ingrisch, Roland Hasibeder, Markus Lange, Sandra Lavorel, Michael Bahn, Gerd Gleixner

**Affiliations:** ^1^ Max Planck Institute for Biogeochemistry Jena Germany; ^2^ Institute of Agro‐Environmental and Forest Biology CNR Italy Porano (TR) Italy; ^3^ Institute of Ecology University of Innsbruck Innsbruck Austria; ^4^ Laboratoire d'Ecologie Alpine UMR 5553 CNRS Université Joseph Fourier Grenoble Cedex 9 France

**Keywords:** ^13^C pulse labelling, below‐ground carbon allocation, carbohydrates, land abandonment, nitrogen uptake, NLFA, PLFA, resilience, resistance, stress tolerance

## Abstract

1. Mountain grasslands have recently been exposed to substantial changes in land use and climate and in the near future will likely face an increased frequency of extreme droughts. To date, how the drought responses of carbon (C) allocation, a key process in the C cycle, are affected by land‐use changes in mountain grassland is not known.
2. We performed an experimental summer drought on an abandoned grassland and a traditionally managed hay meadow and traced the fate of recent assimilates through the plant–soil continuum. We applied two ^13^ CO_2_ pulses, at peak drought and in the recovery phase shortly after rewetting.
3. Drought decreased total C uptake in both grassland types and led to a loss of above‐ground carbohydrate storage pools. The below‐ground C allocation to root sucrose was enhanced by drought, especially in the meadow, which also held larger root carbohydrate storage pools.
4. The microbial community of the abandoned grassland comprised more saprotrophic fungal and Gram(+) bacterial markers compared to the meadow. Drought increased the newly introduced AM and saprotrophic (A+S) fungi:bacteria ratio in both grassland types. At peak drought, the ^13^C transfer into AM and saprotrophic fungi, and Gram(−) bacteria was more strongly reduced in the meadow than in the abandoned grassland, which contrasted the patterns of the root carbohydrate pools.
5. In both grassland types, the C allocation largely recovered after rewetting. Slowest recovery was found for AM fungi and their ^13^C uptake. In contrast, all bacterial markers quickly recovered C uptake. In the meadow, where plant nitrate uptake was enhanced after drought, C uptake was even higher than in control plots.
6. *Synthesis*. Our results suggest that resistance and resilience (i.e. recovery) of plant C dynamics and plant‐microbial interactions are negatively related, that is, high resistance is followed by slow recovery and vice versa. The abandoned grassland was more resistant to drought than the meadow and possibly had a stronger link to AM fungi that could have provided better access to water through the hyphal network. In contrast, meadow communities strongly reduced C allocation to storage and C transfer to the microbial community in the drought phase, but in the recovery phase invested C resources in the bacterial communities to gain more nutrients for regrowth. We conclude that the management of mountain grasslands increases their resilience to drought.

## 1 INTRODUCTION

Extreme drought events may be the biggest climate change‐related threat for the global carbon cycle (Reichstein et al., 2013), and their impacts on mountain ecosystems are highly uncertain (IPCC, 2007, 2012, 2013). In the European Alps, temperature increased twice as fast during the last century than in the remaining northern hemisphere (Auer et al., 2007). Moreover, regional climate models project additional temperature increases that are accompanied by lower precipitation during summer (Gobiet et al., 2014). Therefore, further research to understand the impact of extreme droughts on mountain ecosystems is needed.

Mountain ecosystems are also impacted by socioeconomic changes, which typically lead to changes in land management intensity and land‐use change (MacDonald et al., 2000; Spehn & Körner, 2005; Tasser & Tappeiner, 2002; Vittoz, Randin, Dutoit, Bonnet, & Hegg, 2009). The abandonment of marginal grasslands changes the composition of plant communities and their likely response to environmental factors. Abandonment also leads to (1) changes in the C dynamics, like lower plant productivity (Schmitt, Bahn, Wohlfahrt, Tappeiner, & Cernusca, 2010), (2) shifts from root to shoot litter inputs (Meyer, Leifeld, Bahn, & Fuhrer, 2012), (3) more fungal‐dominated soil communities (Zeller, Bardgett, & Tappeiner, 2001) and (4) changes in nutrient dynamics, like slower nitrogen (N) cycling in soil (Robson, Lavorel, Clement, & Roux, 2007; Zeller, Bahn, Aichner, & Tappeiner, 2000). Currently, it remains unclear how these altered ecosystems respond to climatic extremes (Bahn, Reichstein, Dukes, Smith, & McDowell, 2014).

To investigate the response of ecosystems to disturbances, such as climate extremes, we have to consider two different factors. On the one hand, the capacity of a system to resist to disturbances, that is, the ability to maintain ecosystem functioning during a perturbation, and on the other hand, its “resilience,” that is, the ability to return to initial ecosystem functioning after a perturbation (Nimmo, Mac Nally, Cunningham, Haslem, & Bennett, 2015; Pimm, 1984). The resistance of a system can be measured directly at maximum stress in comparison with a control (Nimmo et al., 2015). Resilience can be measured only after the stress is released, either as time till the functioning is fully recovered or at a given time point quantifying the remaining stress response (Hodgson, McDonald, & Hosken, 2015; Yeung & Richardson, 2016). Currently, it remains unclear if high resistance that keeps a function active will also lead to faster recovery of this function.

Below‐ground C allocation (BCA) is a key process of the carbon cycle that influences the residence time of C in ecosystems and promotes the ability of plants to recover from disturbances (Brüggemann et al., 2011; Chapin, Schulze, & Mooney, 1990). However, so far the response of BCA to drought is variable. Sometimes BCA decreases (Ruehr et al., 2009), sometimes it remains unchanged (Hasibeder, Fuchslueger, Richter, & Bahn, 2015) and sometimes BCA increases during drought (Barthel et al., 2011; Burri, Sturm, Prechsl, Knohl, & Buchmann, 2014; Huang & Fu, 2000; Palta & Gregory, 1997). It is very likely that drought increases the need of recent assimilates in the roots for maintenance respiration (Barthel et al., 2011), for growth (Burri et al., 2014; Huang & Fu, 2000) and for osmotic adjustment (Hasibeder et al., 2015; Van den Ende, 2013; Vijn & Smeekens, 1999). Often, the enhanced BCA under stress is maintained at the expense of above‐ground C storage (Bahn et al., 2013; Barthel et al., 2011) and either less storage carbohydrates (e.g. starch, fructans) are produced or the storage pools are metabolized to sucrose that is needed for transport and for the formation of below‐ground C storages (Benot et al., 2013; Brüggemann et al., 2011). In consequence, compound‐specific investigations are needed to better understand the underlying mechanisms.

However, BCA also influences the soil‐microbial activity and community structure and their feedbacks to the plant community (Bahn et al., 2013; Bardgett, Bowman, Kaufmann, & Schmidt, 2005; Bardgett, de Deyn, & Ostle, 2009; Chapin et al., 2009; Gleixner, 2013; Kuzyakov, 2010). First of all, the microbial community facilitates plant access to soil‐derived nutrients (e.g. nitrogen and phosphorus) that are necessary for plant regrowth after disturbance. However, the role of individual parts of the microbial community has to be differentiated. Arbuscular mycorrhiza (AM) fungi can improve plant water uptake during drought and may consequently contribute to plant resistance to drought (Allen, 2007). Greater fungal biomass, frequently observed in abandoned grasslands compared to managed grasslands (Grigulis et al., 2013; Zeller et al., 2000, 2001), enhances the resistance to drought (de Vries et al., 2012; Fuchslueger, Bahn, Fritz, Hasibeder, & Richter, 2014a; Schimel, Balser, & Wallenstein, 2007). On the other hand, bacteria‐dominated communities may contribute more to the resilience of plant communities because of their faster response time and higher growth rate (de Vries et al., 2012). Gram‐negative bacteria are, for example, directly linked to the flow of root exudates (Bahn et al., 2013; Denef, Roobroeck, Manimel Wadu, Lootens, & Boeckx, 2009; Kramer & Gleixner, 2008). In contrast, Gram‐positive bacteria, which additionally feed on soil organic matter (Bai, Liang, Bodé, Huygens, & Boeckx, 2016; Kramer & Gleixner, 2008; Mellado‐Vázquez et al., 2016), may be more resistant to drought (Lennon, Aanderud, Lehmkuhl, & Schoolmaster, 2012; Schimel et al., 2007) than Gram‐negative bacteria and may even benefit from pulses of organic matter induced by drought (Fuchslueger et al., 2014a). Isotopic pulse‐chase experiments provide the experimental platform to determine the interactions between plant and soil‐microbial communities (Mellado‐Vázquez et al., 2016).

Drought events (Fuchslueger et al., 2014a; Hasibeder et al., 2015) and grassland management (Grigulis et al., 2013; Schmitt et al., 2010), taken independently, affect C and N cycling in mountain grasslands. However, the combined effects of drought and grassland management intensity and how they affect the resistance and resilience of the grassland community are not well known. Here, we experimentally simulated early summer drought for two mountain grassland communities from an abandoned grassland and a managed hay meadow in an common garden experiment and assessed changes in plant C allocation and plant–soil C transfer using a ^13^C pulse‐labelling approach at peak drought (resistance labelling) and in the recovery phase (resilience labelling). The main focus of this study was to understand (1) how drought affects the C partitioning between storage and transport carbohydrates, (2) how BCA and C transfer to the microbial community respond during and after drought and (3) how land use affects C allocation and its resistance and resilience to drought. We hypothesize that BCA in abandoned grasslands will have greater resistance to drought than hay meadows, due to its comparatively lower productivity and its fungal‐dominated microbial community. We furthermore hypothesize that abandoned grasslands will have lower resilience than managed grasslands, because managed meadows and their microbial communities are better adapted to recover from disturbance. Thus, we expect that after rewetting plant C transfer to the rhizosphere recovers more quickly in the managed compared to the abandoned grassland.

## 2 MATERIALS AND METHODS

### 2.1 Site

The study site is located near Neustift in the Stubai valley in the Austrian Central Alps and is described with its different land‐use types by Schmitt et al. (2010). Briefly, both grassland types considered here, an abandoned grassland (1,970–2,000 m a.s.l.; 47°07′31″N, 11°17′24″E) and a hay meadow (1,820–1,850 m a.s.l.; 47°07′45″N, 11°18′20″E), are situated at a southeast exposed hillside with similar inclination (19°–20°), average annual temperature (3°C), annual precipitation (1,097 mm) and soil type (dystric cambisol). The abandoned grassland has been unmanaged for more than 30 years and has a *Seslerio‐Caricetum* vegetation community, which is invaded by dwarf shrubs (e.g. *Calluna vulgaris* and *Vaccinium myrtillus*). The meadow is cut once per year at peak biomass in early August and manured every 2–3 years and has a *Trisetum flavescentis* vegetation community consisting of perennial grasses and forbs (Bahn, Schmitt, Siegwolf, Richter, & Bruggemann, 2009). Spring biomass is higher in the meadow (190–313 g/m^2^) than in the abandoned grassland (106–215 g/m^2^), while peak biomass in summer is similar for both grassland types (*c*. 400 g/m^2^; Schmitt et al., 2010). Abandoned grassland soil has higher contents of SOM, extractable organic N and NH_4_^+^ than meadow soil, which instead has a higher NO_3_^−^ content and a lower C:N ratio (Fuchslueger et al., 2014b). Bulk density (Meyer et al., 2012) as well as total C and N contents (Zeller et al., 2001) and root N concentrations (Bahn, Knapp, Garajova, Pfahringer, & Cernusca, 2006) are higher in the meadow than in the abandoned grassland. Higher fungal biomass was reported for the abandoned grassland compared to meadow (Grigulis et al., 2013; Zeller et al., 2001).

### 2.2 Experimental setup and labelling

For both sites, abandoned and meadow, intact vegetation‐soil monoliths with *c*. 30 cm soil depth and 25 cm diameter were taken in summer 2013. The monoliths were transferred into stainless steel cylinders with collection space for leachates at the bottom (deep seepage collectors, DSCs; Obojes et al., 2015) and were embedded together in the soil at the meadow site (Ingrisch et al., 2017). In this commonly applied approach, the diameter and the depth of the monoliths might exclude some species present at the two sites and might damage roots as well as mycorrhizal networks. To overcome the latter effect, we preincubated the monoliths for 1 year at the experimental site. While the monoliths probably did not cover all plant species present in these very diverse grasslands (Spehn & Körner, 2005), we are confident that we sampled representative subsets of both grassland communities. In spite of the potential drawbacks, this study design allowed us to investigate the drought response of both land‐use types at most comparable conditions, using a randomized block design with replicated drought and control treatments for both land‐use types (Figure S1).

In total, 24 monoliths were utilized in this study, to perform two labelling campaigns with three replicates for each land‐use type and each control/drought treatment (2 × 3 × 2 × 2). Monoliths from the abandoned grassland held about 70% grasses, 26% forbs, 1% legumes and 3% dwarf shrubs, while monoliths from the meadow held about 54% grasses, 44% forbs, 2% legumes and no dwarf shrubs. To prevent a possible inflow of runoff water into the monoliths, the surface level of the DSC cylinders was 2 cm elevated relative to the surrounding soil surface. All monoliths were preincubated over winter on‐site and the experiment was started on 21 May 2014 by simulating early summer drought. Six rain‐out shelters with a base area of 3 × 3.5 m and 2.5 m height, covered by light‐ and UV‐B permeable plastic foil (Lumisol clear AF, Folitec, Westerburg, Germany, light transmittance *c*. 90%), were installed overall monoliths. Air ventilation was facilitated by leaving the shelters open at the bottom (<0.5 m above‐ground) and at the top of the face sides. Monoliths of control treatments were watered manually during rain exclusion, exceeding natural precipitation by 35% for the abandoned grassland and by 43% for the meadow. The amount of water added was adjusted according to soil moisture measurements to avoid water limitation for controls and to compensate for the increased evapotranspiration under the rain‐out shelters as well as naturally occurring drought (Ingrisch et al., 2017). Soil temperature (S‐TMB sensor and HOBO Micro Station H21‐002 data logger; Onset Computer Corporation, Bourne, MA, USA) and soil water content (Decagon EC‐5, 5TM, 5TE (combined SWC, Temperature), logger Em50; Decagon Devices, Pullmann, WA, USA) were monitored continuously in the main rooting horizon on subplots for each land‐use type and treatment. On 21 June 2014, the first ^13^C pulse‐labelling campaign on 12 monoliths started, and after finishing on 28th June 2014, the drought simulation was stopped exactly after 5.5 weeks. The rain‐out shelters were removed and 50 mm of water was added to all monoliths, which was enough to obtain leachates at the bottom of all DSCs. At the end of rewetting, 20 mg of water‐dissolved KNO_3_ with 10% ^15^N (2 mg ^15^N and 100 ml water per monolith) was distributed equally on the soil of the remaining 12 unlabelled monoliths, which were later used for the second ^13^C pulse‐labelling campaign. After a recovery phase of around 2½ weeks, the recovery labelling was started on 16 July 2014.

The ^13^C pulse labellings were done always on four monoliths per day, representing both land‐use types (abandoned grassland/meadow) and both precipitation treatments (control/drought). The resistance labelling was done on three consecutive days (21 till 23 June) with high radiation. Due to weather conditions, this was not possible for recovery labelling, which was conducted on 16, 18 and 19 July. The pulse labelling was performed similarly as described by Bahn et al. (2009, 2013) and Hasibeder et al. (2015). Briefly, a cylindrical and transparent Plexiglas chamber with 25 cm diameter and 50 cm height was placed on the top of the monoliths with a rubber gasket in between the chamber and the DSC. Elastic bands were used to fix the chamber on external anchor points to ensure gas tightness. Fans and tubes connected to a pump that circulated water cooled with ice packs did air circulation and temperature control, respectively. During the pulse labelling, we monitored the internal air temperature (shaded sensor), CO_2_ concentration (Licor 840A; Lincoln, NE, USA) and ^13^C isotope ratio of CO_2_ (Picarro G2101i Analyzer; Picarro Inc., Santa Clara, CA, USA). Solar radiation was measured outside the chamber using a PAR quantum sensor (PQS 1; Kipp & Zonen, Delft, the Netherlands). Pulse labelling was done under comparable light conditions on mostly clear days between 9:45 and 14:45 CET. Highly enriched ^13^CO_2_ (99.27 atom‐% ^13^C; CortecNet, Voisins‐Le‐Bretonneux, France) was added to achieve *c*. 50 atom‐% ^13^C in chamber CO_2_ with a concentration range of 400–800 ppm during a labelling time of 75 min.

### 2.3 Sampling

Plant and soil samples were collected 1.5 hr, 5 hr, 1 day, 2 days, 3 days and 5 days after the pulse labelling. Natural abundance samples were collected from separate monoliths on 26th and 27th June, representing each land‐use type and treatment (averaged for later analysis). From a surface of around 10 cm^2^, shoot material was cut around 0.5 cm above soil, and soil samples from the first 7 cm were taken directly below the cut surface using a stainless steel tube with 3 cm inner diameter. The metabolic activity of fresh shoots was immediately stopped using microwaves (Popp et al., 1996) and the treated shoots were stored on ice packs for transport. Roots were removed from the soil while carefully sieving the soil to 2 mm. Soil for phospholipid fatty acid (PLFA) and neutral lipid fatty acid (NLFA) analysis was directly frozen in liquid N_2_ and stored at −20°C until further preparation. Subsamples of frozen soil were used to determine the soil water content gravimetrically, by weighing the soil before and after drying for 48 hr at 105°C. The soil water content was calculated as average overall sampling times for each monolith. Roots were washed from remaining soil and dead and coarse roots (diameter >2 mm) were removed. Fine root samples were portioned into two subsamples. One subsample was treated in the same way like shoot samples, and the other one was kept moist with wet paper towels until root respiration measurements. If total root biomass was low, no subsample for root respiration measurements was taken. Microwaved shoot and root samples were dried at 60°C for 72 hr on the same day. Root biomass was directly estimated from the dry mass of all root samples from one monolith. For shoot biomass, all monoliths were harvested completely at the end of each sampling campaign and the total dry mass per monolith was determined. All plant material was ball milled for further analyses (MM200; Retsch GmbH, Haan, Germany).

### 2.4 Root respiration measurements

Root respiration was measured directly in the field. About 0.2 to 1.2 mg fresh roots were incubated in a 100 ml Erlenmeyer flask at 15 ± 1°C in a water bath (Hasibeder et al., 2015). Five gas samples were collected, one immediately after closing the flask and the other four after 7, 20, 40 and 60 min. The concentration of CO_2_ and the ^13^C isotope composition were analysed by isotope ratio mass spectrometry (IRMS; Delta^+^ XL; Thermo Fisher Scientific, Bremen, Germany). All gas samples were analysed at the latest 2 weeks after sampling.

### 2.5 Isotopic composition of plant samples and carbohydrates

The ^13^C and ^15^N contents of plant samples were analysed by elemental analysis (EA)‐IRMS (EA 1100, CE Elantech, Milan, Italy; coupled to a Delta+ IRMS; Finnigan MAT, Bremen, Germany). For carbohydrate analysis, 30 mg of plant powder was weighed and water soluble sugars were extracted using the method of Wild, Wanek, Postl, and Richter (2010), as modified by Mellado‐Vázquez et al. (2016). In brief, 3 × 1.5 ml of boiling bidistilled water was added to the plant material and extraction was carried out for 3 × 10 min at 85°C at 1,050 rpm in a horizontal shaker (Thermomixer comfort, Eppendorf AG, Hamburg, Germany). The samples were centrifuged and the combined supernatant was filtered with 0.45 μm cellulose membrane filters (MULTOCLEAR 0.45 μm RC 13 mm; CS‐Chromatographie Service GmbH, Langerwehe, Germany) and transferred to anion and cation exchange cartridges (Dionex OnGuard II A and H 1.0 cc cartridges; Thermo Scientific, Sunnyvale, CA, USA) to remove ionic components. The neutral fraction was analysed by high‐performance liquid chromatography (HPLC)‐IRMS (Dionex UltiMate 3000 UHPLC coupled via a LC‐IsoLink system to a Delta V Advantage IRMS; Thermo Fisher Scientific) on a NUCLEOGEL SUGAR 810 Ca^2+^ column (Macherey‐Nagel GmbH & Co. KG, Düren, Germany) at 80°C with a flow of 0.5 ml/min bidistilled water (Hettmann, Brand, & Gleixner, 2007). Fructans were mostly visible as one large peak at the beginning of the chromatogram (Benot et al., 2013) and their identity was confirmed after hydrolyses with inulinase from *Aspergillus niger* (Sigma‐Aldrich Chemie GmbH, Munich, Germany) using the HPLC‐IRMS. Starch was analysed from the remaining pellets of the sugar extraction. The pellet was washed with a methanol:chloroform:water mixture (12:3:5, by volume) to remove potentially remaining sugars and lipids. The starch was digested with heat stable α‐amylase (Göttlicher, Knohl, Wanek, Buchmann, & Richter, 2006; Richter et al., 2009) and finally resulting gluco‐oligomer solution was measured after drying at 40°C by EA‐IRMS (see above).

### 2.6 Neutral and phospholipid fatty acid content and C isotope composition

Neutral and PLFAs were extracted from frozen soil samples using the modified method of Bligh and Dyer (1959), according to Kramer and Gleixner (2006). In this study, total lipids were extracted from *c*. 5 g of bulk soil using pressurized solvent extraction (SpeedExtractor E‐916; Büchi Labortechnik AG, Flawil, Switzerland) with a mixture of methanol, chloroform and 0.05 M K_2_HPO_4_ buffer (2:1:0.8, by volume; pH 7.4). The soil samples were mixed with precombusted quartz sand and transferred into 40 ml stainless steel extraction cells, a recovery standard (1,2‐Dinonadecanoyl‐sn‐Glycero‐3‐Phosphatidylcholine; Larodan Fine Chemicals AB, Malmö, Sweden) was added on top (recovery rate: 93 ± 27%, *n* = 52) and the extraction was carried out at 70°C and 120 bar for 3 × 10 min. The pressurized solvent extraction yielded similar amounts of PLFAs compared to the established method (Kramer & Gleixner, 2006) if the extraction was done near room temperature at 40°C (Figure S2). Using 70°C, the extraction efficiency was increased by around 50% on average (Table S1). After extraction, the separated chloroform phase was subjected to silica‐filled solid‐phase extraction (SPE) columns (CHROMABOND SiOH, 2 g, 15 ml; Macherey‐Nagel GmbH & Co. KG) to obtain neutral lipid and phospholipid fractions. Both fractions were hydrolysed and methylated with methanolic KOH and resulting fatty acid methyl esters (FAMEs) were further purified using aminopropyl‐modified SPE columns (CHROMABOND NH_2_, 0.5 g, 3 ml; Macherey‐Nagel GmbH & Co. KG). The FAME C13:0 (Sigma‐Aldrich Chemie GmbH) was added as internal standard to all samples prior to quantification by gas chromatography‐flame ionization detection (GC‐FID).

The PLFAs were analysed on a GC‐FID 7890B with a programmable temperature vapourisation (PTV) injector (Agilent Technologies, Palo Alto, CA, USA) using a DB‐1MS UI column (60 m × 0.25 mm internal diameter × 0.25 μm film thickness; Agilent Technologies) and helium as carrier gas (1.8 ml/min). The temperature programme started at 45°C for 1 min, then increased in a first ramp of 60°C/min to 140°C, held for 0.5 min, followed by a second ramp of 2°C/min until 264°C and a third ramp until 320°C, held for 3 min. Directly after injection, the PTV was heated up from 55°C to 280°C at a rate of 500°C/min.

Neutral lipid fatty acids were quantified on a GC‐FID HP6890 (Agilent Technologies) with constant injector temperature (280°C), using a DB‐1MS column (50 m × 0.32 mm internal diameter × 0.52 μm film thickness, Agilent Technologies) and helium as carrier gas (2 ml/min). The temperature programme started with 140°C for 1 min, followed by a first ramp of 2°C/min until 270°C, held for 6 min and a second ramp of 30°C/min until 340°C, held for 5 min.

Identification of FAMEs was done by comparison of chromatograms with different known FAME mixtures (Supelco 37 Component FAME Mix; Sigma‐Aldrich Chemie GmbH; BR2 and BR4 mixture, Larodan Fine Chemicals AB) and an in house database (Kramer & Gleixner, 2006; Mellado‐Vázquez et al., 2016; Thoms, Gattinger, Jacob, Thomas, & Gleixner, 2010).

Compound‐specific ^13^C isotope analysis of NLFAs and PLFAs was done by GC‐IRMS (GC 7890A with PTV injector; Agilent Technologies; coupled via a Conflo IV/GC IsoLink to a Delta V Plus IRMS; Thermo Fisher Scientific) using a DB‐1MS UI column (60 m × 0.25 mm internal diameter × 0.25 μm film thickness; Agilent Technologies) and helium as carrier gas (1.8 ml/min). Directly after injection, the PTV was heated up from 55°C to 280°C at a rate of 500°C/min. The GC temperature programme started with 45°C for 1 min, then increased in a first ramp of 60°C/min to 140°C (held for 0.5 min), followed by a second ramp of 4°C/min until 283°C (held for 4.9 min) and a third ramp until 320°C (held for 3 min). Concentrations and ^13^C isotope content of identified FAMEs were corrected for the methyl group introduced during derivatization. We used the sum of the PLFAs i14:0, i15:0, a15:0, i16:0, a17:0, i17:0 and br18:0 for Gram‐positive bacteria (Zelles, 1997, 1999); 10Me16:0 and 10Me18:0 for Gram‐positive actinobacteria (Lechevalier, De Bievre, & Lechevalier, 1977; Zelles, 1999) and 16:1ω7 and 18:1ω7 for Gram‐negative bacteria (Zelles, 1997, 1999). The PLFA 18:2ω6,9c was used as marker for saprotrophic fungi (Frostegård & Bååth, 1996; Zelles, 1997) and the NLFA 16:1ω5 as marker for AM fungi (Olsson, 1999). Despite its uncertainty as predictor for AM fungi biomass, the NLFA 16:1ω5 is supposed to be more indicative for AM fungi than the PLFA 16:1ω5, based on previous findings showing that the PLFA 16:1ω5 is closer related to bacteria (Mellado‐Vázquez et al., 2016). Principal component analyses of all PLFA quantified in this study also showed a strong correlation of the PLFA 16:1ω5 with bacterial makers while the supplementary added NLFA 16:1ω5 had an opposite trend, more related to the saprotrophic fungi marker (Figure S3).

### 2.7 Calculation of incorporated ^13^C and ^15^N

For all plant and soil samples, we expressed the ^13^C isotope content as incorporated ^13^C (mg ^13^C/m^2^, μg ^13^C/m^2^ or ng ^13^C/g_dry matter_), which refers to the total amount of ^13^C found in a certain C pool:

$$\text{incorporated}\;{}^{13}C=\frac{\left(\text{atom}{\%}_{\mathrm{labelled}}-\text{atom}{\%}_{\mathrm{unlabelled}}\right)\times C_{\mathrm{pool}}}{100\%}$$

with atom%_labelled_ being the ^13^C atom% of the labelled samples, atom%_unlabelled_ being the ^13^C atom% of natural abundance samples and C_pool_ being the respective C pool (mg C/m^2^ for bulk and carbohydrate data from shoots and fine roots; μg ^13^C/m^2^ or ng C/g_dry matter_ for NLFAs and PLFAs from soil). Incorporated ^15^N of plant samples was calculated in a completely analogous fashion. Root respired ^13^C (μmol ^13^C m^−2^ hr^−1^), which corresponds to the amount of ^13^C released in respired CO_2_ from roots during a certain time, was calculated similar to incorporated ^13^C:

$$\text{root respired}{}^{13}C=\frac{\left(\text{atom}{\%}_{\mathrm{labelled}}-\text{atom}{\%}_{\mathrm{unlabelled}}\right)\times {\text{CO}}_{2,\mathrm{resp}.\mathrm{rate}}}{100\%}$$

with CO_2, resp. rate_ being the respiration rate of CO_2_ (μmol CO_2_ m^−2^ hr^−1^).

### 2.8 Data analyses

For concentration measurements, average values were calculated over the different sampling times after pulse labelling (1.5 hr, 1 day, 3 days and 5 days for carbohydrates and root respired CO_2_; 1 day and 3 days for NLFAs and PLFAs). If necessary, the data were corrected for bulk density differences (Meyer et al., 2012).

For soil‐microbial community, the (A+S)‐fungi:bacteria ratio was calculated by dividing the sum of the AM fungi marker (NLFA 16:1ω5) and the saprotrophic fungi marker (18:2ω6,9) by the sum of all bacterial PLFA markers, similar to the previously used fungi:bacteria ratio (de Vries & Shade, 2013; de Vries et al., 2012; Fuchslueger et al., 2014a).

Total ^13^C uptake was calculated as sum of bulk shoot and bulk root‐incorporated ^13^C directly after labelling (1.5 hr sampling). Total ^15^N uptake was calculated as average overall sampling times because the signal was stable over the experimental time.

All statistical analyses were done using the r 3.3.2 software (R Core Team, 2016). The effects of drought treatment, land‐use type and their interaction on soil water content, fine root biomass, carbohydrate concentrations, NLFA and PLFA concentrations, (A+S)‐fungi:bacteria ratio as well as ^13^C and ^15^N tracer uptake were evaluated for each labelling campaign separately using ANOVA from the R base package and permutational ANOVA from the “lmPerm” package (Wheeler & Torchiano, 2016). We used the standard ANOVA to estimate effect sizes based on *F*‐values and the permutational ANOVA to obtain exact *p*‐values. Permutation tests do not require assumptions about the statistical distribution and are more sensitive with small sample sizes (Ernst, 2004). Time series (in hours after pulse labelling) of ^13^C tracer data were tested for each labelling campaign separately for the effects of drought, land‐use type, sampling time and their interaction using linear mixed‐effect models from the “lme4” package (Bates, Maechler, Bolker, & Walker, 2015). In the mixed‐effects models treatment, land use and sampling time (as factor) were set as fixed effects, while rain‐out shelter and monolith were set as random effects. All models were assessed for violations of normality, heteroscedasticity and independency, and if necessary, ^13^C tracer data were log (+1) or square root (+1) transformed.

## 3 RESULTS

### 3.1 Drought effects on plant C allocation and recovery

At the resistance labelling, drought reduced the assimilation of ^13^C in both grassland types (Table 1, Table S2). This reduction was stronger in the meadow than in the abandoned grassland. Simultaneously, the concentrations of storage carbohydrates, that is, fructan and starch, decreased in the shoots of both communities, and led to a strong increase in root sucrose (Table 1, Table S2). This increase was stronger in the meadow than in the abandoned grassland. The root carbohydrate storage was unaffected by drought, but larger storage pools were found in the meadow.

**Table 1:** Soil water content, fine root biomass, total ^13^C and ^15^N uptake, root respiration rate, concentrations of plant carbohydrates, concentrations of soil‐microbial marker lipids and (A+S)‐fungi:bacteria ratio for control/drought treatments of abandoned grassland and meadow (*M* ± *SE* of *n* = 3 monoliths) at the resistance labelling (peak drought) and the resilience labelling (recovery phase)

Labelling	Parameter	Unit	Abandoned		Meadow	
			Control	Drought	Control	Drought
Resistance	*General*					
	SWC	mass‐%	38 ± 3	22 ± 1	38 ± 1	14 ± 1
	Fine roots	g/m^2^	348 ± 35	352 ± 46	228 ± 42	252 ± 9
	Total ^13^C uptake	mg/m^2^	742 ± 59	632 ± 171	1,165 ± 255	785 ± 129
	Root resp. CO_2_	nmol g_dm_^−1^ s^−1^	2.38 ± 0.01^a^	1.69 ± 0.09^a^	3.25 ± 0.16^a^	3.34^b^
	*Carbohydrates*					
	Shoot sucrose	mg_C_/g_dm_	20.9 ± 2.4	22.1 ± 2.4	14.7 ± 1.5	16.8 ± 0.9
	Shoot fructan		38.3 ± 6.2	26.3 ± 3.3	34.7 ± 2.9	30.2 ± 3.9
	Shoot starch		4.5 ± 0.1	4.8 ± 0.2	8.6 ± 1.9	3.2 ± 0.7
	Root sucrose		3.0 ± 0.5	6.2 ± 0.8	5.5 ± 1.0	11.2 ± 1.4
	Root fructan		19.8 ± 1.2	16.5 ± 2.3	29.1 ± 2.4	32.3 ± 2.9
	Root starch		4.3 ± 0.4	6.2 ± 2.4	14.5 ± 3.6	10.1 ± 1.4
	*Micro*‐*organisms*					
	AM fungi	mg_C_/m^2^_0‐7 cm_	670 ± 176	1,040 ± 123	725 ± 366	808 ± 263
	Sapro. fungi		351 ± 60	385 ± 53	224 ± 19	228 ± 8
	Gram(−) bacteria		1,339 ± 193	1,433 ± 108	1,200 ± 238	1,110 ± 58
	Gram(+) bacteria		1,197 ± 188	1,241 ± 97	884 ± 138	863 ± 33
	Actinobacteria		365 ± 55	374 ± 35	400 ± 81	375 ± 9
	(A+S)‐F:B	–	0.34 ± 0.03	0.47 ± 0.04	0.35 ± 0.08	0.45 ± 0.11
Resilience	*General*					
	SWC	mass‐%	43 ± 5	36 ± 1	37 ± 2	37 ± 1
	Fine roots	g/m^2^	264 ± 18	333 ± 13	237 ± 14	219 ± 11
	Total ^13^C uptake	mg/m^2^	1,293 ± 122	1,355 ± 108	998 ± 189	1,381 ± 66
	Root resp. CO_2_	nmol g_dm_^−1^ s^−1^	2.38 ± 0.38	2.19 ± 0.19	2.90 ± 0.07	2.72 ± 0.40
	Plant ^15^N uptake^c^	mg/m^2^	1.4 ± 0.1	1.6 ± 0.1	1.8 ± 0.3	3.1 ± 0.5
	*Carbohydrates*					
	Shoot sucrose	mg_C_/g_dm_	16.4 ± 1.9	16.0 ± 2.1	13.3 ± 2.1	10.5 ± 1.8
	Shoot fructan		57.7 ± 2.0	43.8 ± 7.9	45.6 ± 4.5	40.8 ± 4.4
	Shoot starch		4.2 ± 0.5	4.3 ± 0.9	6.1 ± 0.2	7.0 ± 1.3
	Root sucrose		2.8 ± 0.4	5.1 ± 1.6	7.6 ± 1.7	5.5 ± 1.0
	Root fructan		21.1 ± 2.5	18.9 ± 3.8	34.6 ± 1.7	29.1 ± 2.1
	Root starch		2.7 ± 0.1	3.5 ± 0.7	4.8 ± 0.5	3.6 ± 0.7
	*Micro*‐*organisms*					
	AM fungi	mg_C_/m^2^_0‐7 cm_	764 ± 303	369 ± 51	817 ± 467	213 ± 68
	Sapro. fungi		308 ± 42	333 ± 92	202 ± 33	214 ± 15
	G(−) bacteria		1,094 ± 91	1,227 ± 221	1,037 ± 276	1,169 ± 147
	G(+) bacteria		1,079 ± 106	1,099 ± 220	807 ± 186	1,073 ± 174
	Actinobacteria		326 ± 39	328 ± 60	379 ± 106	423 ± 64
	(A+S)‐F:B	–	0.43 ± 0.14	0.28 ± 0.03	0.47 ± 0.17	0.16 ± 0.01

(A+S)‐F:B, (arbuscular mycorrhiza + saprotrophic) fungi:bacteria ratio; G(‐/+), Gram‐negative/positive; resp., respired; Sapro., saprotrophic; SWC, soil water content.

a Only two replicates could be measured.

b Only one replicate could be measured.

c The ^15^N addition was only done on monoliths used for the resilience labelling, plant ^15^N uptake is the sum of shoot‐ and root‐incorporated ^15^N.

**Figure 1:**
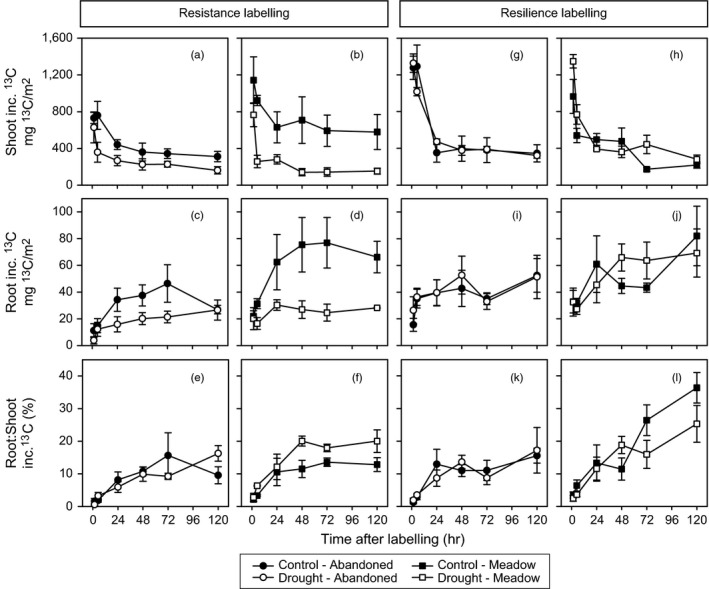
^13^C tracer dynamics in bulk shoots and roots as well as the root to shoot ^13^C ratio over time from abandoned grassland (a, c, e, g, i, k/circles) and meadow (b, d, f, h, j, l/squares) control (closed symbols) and drought (open symbols) monoliths; after the resistance (a–f) and the resilience (g–l) ^13^C pulse labelling. Error bars show ± *SE* (*n* = 3); inc. ^13^C, incorporated ^13^C

**Figure 2:**
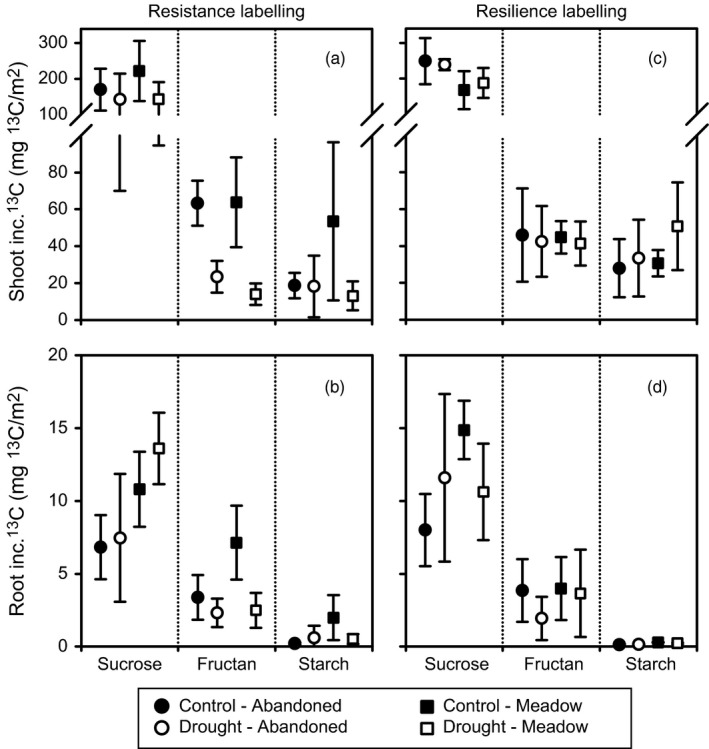
Average ^13^C tracer incorporation into plant shoot (a, c) and root (b, d) carbohydrates of control (closed symbols) and drought (open symbols) monoliths from the abandoned grassland (circles) and the meadow (squares); after the resistance (a, b) and the resilience (c, d) ^13^C pulse labelling. Dotted lines separate amongst the three investigated carbohydrates (sucrose, fructan and starch). Error bars show ± *SE* (*n* = 3); inc. ^13^C, incorporated ^13^C

Drought also reduced the ^13^C tracer dynamics in shoots and roots of both grassland types (Figure 1a–d, Table S3). The observed reductions were larger in the meadow than in the abandoned grassland. In drought treatments, the ^13^C tracer declined faster with time in the shoots and increased less in the roots. The initial label uptake into shoots mainly reflected the high ^13^C incorporation into sucrose (Figure 2a), which was not significantly affected by drought in both grassland types (Table S3) and declined exponentially (Figures S4 and S5). After 24 hr, the shoot tracer dynamics reflected mainly the ^13^C incorporation into shoot storage carbohydrates. The ^13^C content of starch decreased over time, like sucrose, but increased in fructans suggesting that shoot fructans have a much smaller turnover than starch. Drought strongly reduced ^13^C incorporation into the shoot carbohydrate storages of both grassland types, but the ^13^C incorporation into starch of the abandoned grassland was less affected compared to the meadow (Figure 2a, Figures S4 and S5, Table S3), which confirmed the results of the carbohydrate concentrations.

The ^13^C tracer dynamics of root carbohydrates was only little affected by drought at the resistance labelling (Figure 2b, Table S3, Figures S4 and S5). In the meadow, drought reduced the ^13^C incorporation into root storage carbohydrates. In contrast, on the abandoned grassland, no effect or even a slight increase in ^13^C of root starch was observed. Root sucrose had a slower turnover in both grassland types leading to a to higher ^13^C incorporation after 5 days from labelling (Figures S4 and S5, Table S3). This slowdown of ^13^C tracer dynamics in root sucrose was confirmed by the mean residence times (Table S4), but the effect was only significant for the abandoned grassland. Remarkably, the relative amount of ^13^C that was transferred from above‐ to below‐ground, measured by the root to shoot ratio of ^13^C incorporation, was not reduced by drought in both grassland types (Figure 1). In fact, this ratio increased over time in the meadow under drought (Figure 1e,f) and the proportion of ^13^C from the labelling pulse that was found in root sucrose was higher than in controls (Figure S6).

At the resilience labelling, the majority of parameters considered in this study completely recovered and the total ^13^C uptake was already exceeding the control values, especially in the meadow (Table 1, Figures 1 and 2, Tables S2 and S3). The shoot fructan concentrations still not completely recovered for both grassland types. A legacy effect of drought was also visible in root sucrose and root starch. Both carbohydrates were increased in the abandoned grassland and decreased in meadow. Moreover, the previous drought treatment significantly increased the fine root biomass of the abandoned grassland, leading to higher root biomass in comparison with the meadow. The root respiration rate recovered for both grassland types but was generally higher in the meadow. Recovering meadow roots also respired more ^13^CO_2_ (Figure S7). Most interestingly, the plant ^15^N label uptake was increased in the recovery phase, especially in the meadow (Table 1, Table S2).

Furthermore, the ^13^C tracer dynamics in shoots and roots (Figure 1g–j, Table S3) and the shoot carbohydrate ^13^C incorporation (Figure 2c, Table S3) recovered completely. Only the mean residence time of shoot sucrose was still lower in previously drought‐treated meadow (Table S4, Figure S5). The ^13^C incorporation in root sucrose of both grassland types responded slightly different at the resilience labelling (Figure 2d). It was increased for drought treatments in the abandoned grassland, while it was decreased in the meadow (Table S3). Consequently, a smaller proportion of ^13^C from the labelling pulse was found in root sucrose from the recovering meadow community (Figure S6). Overall, at the resilience labelling, BCA was higher in the meadow compared to the abandoned grassland, as more label was found in meadow roots over the course of time (Figure 1i,j, Table S3) and the root:shoot ^13^C incorporation was higher in the meadow (Figure 1k,l, Table S3), while less label was found in bulk shoots and shoot sucrose (Figures 1g,h and 2, Table S3) from the meadow.

### 3.2 Drought effects on C transfer to soil‐microbial community and recovery

**Figure 3:**
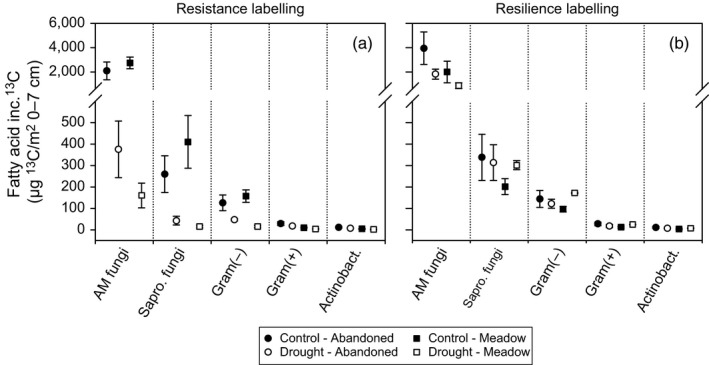
Average ^13^C tracer incorporation in marker fatty acids for arbuscular mycorrhiza fungi (AM fungi), saprotrophic fungi (Sapro. fungi), Gram‐negative bacteria (Gram(−)), Gram‐positive bacteria (Gram(+)) and actinobacteria (Actinobact.), extracted from soil cores from 0 to 7 cm depth of control (closed symbols) and drought (open symbols) monoliths from the abandoned grassland (circles) and the meadow (squares); after the resistance (a) and the resilience (b) ^13^C pulse labelling. Dotted lines separate amongst the five different microbial groups. Error bars show ± *SE* (*n* = 3); inc. ^13^C, incorporated ^13^C

The abandoned grassland held more saprotrophic fungi and Gram‐positive bacteria than the meadow, and this was barely affected by drought (Table 1, Table S2, Figure S3). At the resistance labelling, drought increased the content of AM fungi marker in the abandoned grassland by about 55% on average, but as the variability in this marker is usually high (Olsson, 1999), the effect was insignificant. Nonetheless, the (A+S)‐fungi:bacteria ratio was significantly increased by drought in both grassland types (Table 1, Table S2), although the uptake of recent assimilated plant C by AM fungi and saprotrophic fungi was reduced (Figure 3a, Table S3, Figure S8). However, root‐associated Gram‐negative bacteria received less plant‐derived C in both grassland types under drought. The reductions of ^13^C uptake were consistently stronger in the soil‐microbial community of the meadow compared to the abandoned grassland.

At the resilience labelling, all microbial groups had completely recovered from drought, except for the AM fungi, which had significantly reduced marker concentrations in both grassland types (Table 1, Table S2). Correspondingly, the (A+S)‐fungi:bacteria ratio was significantly reduced by drought and rewetting. Also, the ^13^C incorporation into the AM fungi marker was still reduced, whereas the other microbial groups recovered their label uptake (Figure 3, Table S3, Figure S8). Only in the drought‐treated meadow, the ^13^C uptake was strongly increased in Gram‐negative bacteria and Gram‐positive bacteria including actinobacteria, which was also mirrored by a higher variability in the PLFA composition in the meadow (Figure S3).

## 4 DISCUSSION

**Figure 4:**
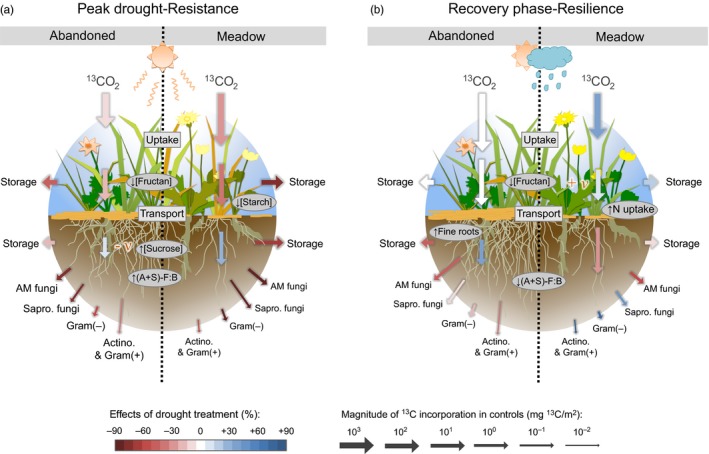
Overview of the effects of drought on ^13^C tracer uptake, allocation in plants and transfer to soil microbes (a) at peak drought (resistance labelling) and (b) in the recovery phase (resilience labelling), in abandoned grassland and meadow. The arrows represent the amount of ^13^C uptake and ^13^C incorporation into different pools following the ^13^C pulse labelling, with the width of the arrow indicating different size classes as determined by the magnitude of ^13^C incorporation in controls, and the length of the arrow describing the relative differences in controls within each size class, so that the comparison between both land use types and labellings is possible. The effects of the drought treatment are expressed separately by a colour gradient indicating the change relative to the control value (red: reduced ^13^C incorporation, white: no change, blue: increased ^13^C incorporation). Shoot and root sucrose pools were used as proxy for transport to the below‐ground (central arrows), with +ν/‐ν indicating higher/lower turnover of ^13^C tracer in drought monoliths. All arrows for plant carbohydrates and soil‐microbial markers represent average values of ^13^C tracer dynamics. Oval boxes show additional information not related to the ^13^C tracer flux and drought‐related changes in pool sizes or biomasses. Actino., actinobacteria; AM, Arbuscular mycorrhiza; (A+S)‐F:B, ratio of AM + saprotrophic fungi to bacteria; Gram(+/−), Gram‐positive/negative bacteria

Our study demonstrates that BCA and plant‐microbial interactions of the managed and abandoned grassland differed in their response to drought and rewetting, and thus highlights the important role of land management for the resistance and resilience of marginal grasslands to climate extremes. In addition, our analyses confirmed that the meadow and the abandoned grassland differed in their initial properties (Figure 4, Table 1). The abandoned grassland held more root biomass, similar as observed by (Bahn et al., 2006), and higher shoot sucrose concentrations, whereas the meadow had higher concentrations of root sucrose and the root storage sugars starch and fructan. This suggests that the abandoned grassland invests in root growth to access soil resources, whereas meadows store resources in roots to facilitate regrowth after cutting. The microbial community of the abandoned grassland held more markers of saprotrophic fungi and Gram‐positive bacteria, which likely benefit from root turnover (Meyer et al., 2012).

Drought affected both grassland plant communities in a similar way (Figure 4a, Table 1). Above‐ground C uptake and storage were reduced and a higher proportion of label was transferred below‐ground. This increase in BCA was stronger in the meadow than in the abandoned grassland. However, recently assimilated C was neither stored in the roots, nor used for growth, nor transferred to the rhizosphere, but remained in the roots as sucrose. As a consequence, the amount of tracer that was transferred to root associated (A+S) fungi and Gram‐negative bacteria strongly decreased and led to a decoupling of plant roots and soil micro‐organisms. This decoupling was weaker in the fungal‐dominated microbial community of the abandoned grassland than in the meadow, although the overall (A+S) fungi:bacteria ratio increased in both grassland types. This suggests that plant communities with conservative species and fungal‐dominated microbial communities are less affected by drought than plant communities with exploitative species and bacterial‐dominated microbial communities.

Our findings are supported by Bahn et al. (2013), who suggested that under reduced C supply BCA is maintained at the cost of above‐ground storage. Unexpectedly, we found that drought‐induced reductions in above‐ground storage were generally stronger in fructans than in starch pools. Fructans are thought to contribute to drought tolerance (Van den Ende, 2013; Vijn & Smeekens, 1999). Although fructans represented the largest part of water soluble carbohydrates, we did not find a correlation with drought resistance, nor an accumulation of fructans, during drought in our study. We also did not find that the high root sucrose concentrations increased root growth and tracer incorporation into fine roots (Burri et al., 2014; Kahmen, Perner, & Buchmann, 2005), which suggests that the increased BCA is not a result of increased sink demand, but is due to osmotic adjustment of roots (Chaves, Maroco, & Pereira, 2003; Chen & Jiang, 2010; Hasibeder et al., 2015; Sicher, Timlin, & Bailey, 2012). This osmotic role of sucrose is further supported by its low transfer into the rhizospere (Fuchslueger et al., 2014a). The reduced plant‐derived C flow also impacts the soil‐microbial community (Barnard, Osborne, & Firestone, 2013; Fuchslueger et al., 2014a). The overall microbial community composition generally seems less affected by drought (Canarini, Carrillo, Mariotte, Ingram, & Dijkstra, 2016), but a general increase in fungi:bacteria ratios is often observed, which may suggest higher resistance of fungal‐based food webs (de Vries et al., 2012; Fuchslueger et al., 2014a). In the abandoned grassland, the amount of AM fungal markers increased during drought (Table 1) and the label uptake in the AM fungal markers was less reduced than in the meadow (Figure 3, Table S3), which suggests that mainly AM fungi are relatively resistant to drought. Thereby, AM fungi can support water and nutrient uptake by plants during drought (Allen, 2007; Wardle et al., 2004). Overall, this supports our initial hypothesis that strong plant‐fungal, specifically plant‐AM fungal, interactions are the basis for the high resistance of the abandoned grassland to drought.

Reduced ^13^C tracer uptake was also found for the other root associated microbial markers of saprotrophic fungi and Gram‐negative bacteria (Bahn et al., 2013; Balasooriya, Denef, Huygens, & Boeckx, 2012; Denef et al., 2009; Kramer & Gleixner, 2008), but not for Gram‐positive bacteria including the actinobacteria (Figures 3a and 4a, Table S3). This was especially expected for the Gram‐negative bacteria that are directly linked to recent plant C input (Bahn et al., 2013; Bardgett et al., 2005; Mellado‐Vázquez et al., 2016), but not for saprotrophic fungi that are generally more resistant to desiccation than Gram‐negative bacteria (Lennon et al., 2012; Schimel et al., 2007). The non‐significant reduction in label uptake into Gram‐positive (actino)‐bacterial PLFAs is in line with their overall low ^13^C uptake compared to root‐associated microbes (Figure 3), their delayed label incorporation (Bahn et al., 2013; Fuchslueger et al., 2014a; Malik, Dannert, Griffiths, Thomson, & Gleixner, 2015) and their preference for additional C sources like soil organic matter (Bai et al., 2016; Kramer & Gleixner, 2008; Mellado‐Vázquez et al., 2016).

In general, the majority of studied parameters quickly recovered after rewetting, but most interestingly, we also found substantial differences between the two grassland types (Figure 4b, Table 1). The meadow recovered quickly and during recovery from drought, its C uptake was even higher than in controls (see also Ingrisch et al., 2017; for CO_2_ fluxes). This C was either allocated to shoot storage or transferred to the rhizosphere. In the abandoned grassland, the C uptake also recovered quickly, but C allocation to storage and transfer to the rhizosphere were still affected by the drought. The higher amount of root sucrose may have facilitated the growth of fine roots (Table 1 and Table S2; Kahmen et al., 2005; Burri et al., 2014). The higher fine root biomass likely increased nutrient and water access after rewetting, possibly because the establishment of new AM fungal‐root connections needed more time, that is, was not resilient. In contrast, the meadow obviously restored the above‐ground biomass after rewetting, since the total ^13^C uptake (Table 1) and shoot sucrose turnover (Table S4) were increased without a change in BCA (Figure 1). Simultaneously, root exudation increased in the meadow, as the ^13^C tracer uptake significantly increased in all bacteria (Table S3, Figure 4b). As a result, the fast regrowth of exploitative meadow plants (Ingrisch et al., 2017) could be supported by the activation of “priming” bacteria (Canarini & Dijkstra, 2015; Kuzyakov, 2010; Roy et al., 2016; Wardle et al., 2004) that led to changes in the microbial community composition (Figure S3b) and likely facilitated a higher N uptake by plants. Overall, the results support our initial hypothesis that the meadow quickly recovers from drought benefiting from strong bacterial interactions, and thus is highly resilient.

Interestingly, our results do not support the hypothesis that in the recovery phase, bacterial communities are favoured over fungal and especially AM fungal communities, as the decreasing (A+S)‐fungi:bacteria ratio would suggest (Table 1). This decrease mainly was driven by the significant decreased abundance of AM fungi and less by the insignificant increase in bacteria (Table 1 and Table S2). This is in line with the finding that fungal‐based food webs are less resilient than bacterial‐based food webs (de Vries et al., 2012; Meisner, Bååth, & Rousk, 2013). Further research is needed to understand the interactions between microbial and plant communities and how they are affected by land use. For example, the rapid recovery of the meadow may result from a history of regular cutting and fertilization, that increased the abundance of “exploitative” species, which can rapidly regrow and effectively gain nutrients (Grassein et al., 2015; Grigulis et al., 2013). This legacy effect of the management could also lead to changes in the soil‐microbial community composition and function (Hawkes & Keitt, 2015), which would enable better acclimatization of certain microbial groups to environmental fluctuations and thereby increase their resilience to drought. Conversely, the more stable conditions, like in the abandoned grassland, might constrain microbial responses during recovery, and thus decrease the resilience of certain microbial groups, as suggested by the “historical contingencies” concept of Hawkes and Keitt (2015). Hence, high resilience of marginal grasslands seems to be based on both, adaptations of plant functional traits and microbial processes, confirming the importance of plant‐microbial interactions to predicting ecological consequences of climate change.

## 5 CONCLUSIONS

Our results highlight that in addition to plant properties, like carbohydrate storage and below‐ground carbon allocation, plant‐microbial interactions influence the resilience mechanisms of ecosystems. In particular, the role of AM fungi for the resistance of plant communities to drought and the role of bacteria in the recovery phase need further research.

Plant‐microbial interactions likely provided better access to resources at different time points, which led to an inverse relationship between resistance and recovery. Resistant communities, which maintain their functioning during drought stress, have fewer nutrient resources available for recovery. Conversely, plant communities that are used to suffer from regular perturbations invest their resources mainly into fast regrowth after disturbance. Both strategies can yield a high overall resilience of ecosystems.

Land use offers the opportunity to manage plant communities and therefore the resilience of ecosystems. Further studies should consequently address the effects of land use on long‐term resilience, including multiple stress events, to maintain the functioning of the endangered marginal grassland systems in a changing world.

## AUTHORS’ CONTRIBUTIONS

M.B., S.L. and G.G. conceived the ideas; S.K., A.A., J.I., R.H., M.B. and G.G. designed methodology; S.K., A.A., J.I., R.H. and G.G. conducted the experiment and collected the data; S.K., A.A. and M.L. analysed the data; S.K. and G.G. led the writing of the manuscript. All authors contributed critically to the drafts and gave final approval for publication.

## DATA ACCESSIBILITY

Data available from the Dryad Digital Repository: https://doi.org/10.5061/dryad.3s57p (Karlowsky et al., 2017).
