# Supplementary material for: Land use in mountain grasslands alters drought response and recovery of carbon allocation and plant‐microbial interactions
Source: J Ecol. 2017 Dec 20;106(3):1230–43. doi: 10.1111/1365-2745.12910 (PMC5947120; doi:10.1111/1365-2745.12910)
Supplement: Supplementary file 1 [file JEC-106-1230-s001.docx]

**Supporting information**

**TABLE S1** Relative extraction efficiency (mean ± SD) of the new PLFA extraction method using pressurised solvent extraction at 40 °C and 70 °C compared to the established method described by Kramer & Gleixner (2006); measured on the same soil sample from an arable field.

| **PLFA group** | ***n*^a^** | **Relative extraction efficiency compared to established method (%)** | | | | | |
| --- | --- | --- | --- | --- | --- | --- | --- |
|  |  | Pressurised extraction at 40 °C | | | Pressurised extraction at 70 °C | | |
| Linear saturated | 7 | 132 | ± | 12 | 170 | ± | 17 |
| Branched saturated | 15 | 118 | ± | 21 | 165 | ± | 43 |
| Cyclic saturated | 2 | 91 | ± | 8 | 109 | ± | 8 |
| Monounsaturated | 14 | 94 | ± | 14 | 116 | ± | 18 |
| Polyunsaturated | 1 | 90 | ± | ─ | 204 | ± | ─ |
| All PLFAs | 39 | 110 | ± | 22 | 146 | ± | 40 |

^a^*n* is the number of PLFAs in each group; for polyunsaturated PLFAs only 18:2ω6c was detected in sufficient amounts.

**TABLE S2** Combined effects of drought and land use on soil water content, fine root biomass, total ^13^C and plant ^15^N uptake, root respiration rate, concentrations of plant carbohydrates, concentrations of soil microbial marker lipids and (A+S)-fungi:bacteria ratio; at the resistance labelling (peak drought) and the resilience labelling (recovery phase).

| **Labelling** | **Parameter** | **Unit** | **Drought (df =1)** | | | **Land use (df =1)** | | | **Drought × Land use (df =1)** | | |
| --- | --- | --- | --- | --- | --- | --- | --- | --- | --- | --- | --- |
|  |  |  | *F*^a^ | *P_F_*^a^ | *P_exact_*^b^ | *F*^a^ | *P_F_*^a^ | *p_exact_*^b^ | *F*^a^ | *P_F_*^a^ | *P_exact_*^b^ |
| **Resistance** | *General* |  |  |  |  |  |  |  |  |  |  |
|  | SWC | mass-% | 169.0 | **<0.001** | **<0.001** | 6.2 | **0.038** | **0.041** | 6.1 | **0.039** | **0.042** |
|  | Fine roots | g/m^2^ | 0.1 | 0.718 | 0.637 | 9.4 | **0.016** | **<0.001** | 0.1 | 0.783 | 0.726 |
|  | Total ^13^C uptake | mg/m^2^ | 2.1 | 0.185 | ***0.052*** | 2.9 | 0.126 | **0.011** | 0.6 | 0.448 | 0.354 |
|  | *Carbohydrates* |  |  |  |  |  |  |  |  |  |  |
|  | Shoot sucrose | mg_C_/g_dm_ | 0.8 | 0.411 | 0.300 | 9.2 | **0.016** | **<0.001** | 0.1 | 0.806 | 0.735 |
|  | Shoot fructan |  | 3.7 | 0.092 | **0.008** | 0.0 | 0.975 | 0.970 | 0.8 | 0.408 | 0.263 |
|  | Shoot starch |  | 6.5 | **0.034** | **<0.001** | 1.6 | 0.241 | 0.183 | 8.7 | **0.019** | **<0.001** |
|  | Root sucrose |  | 20.4 | **0.002** | **<0.001** | 14.3 | **0.005** | **<0.001** | 1.7 | 0.229 | 0.180 |
|  | Root fructan |  | 0.0 | 0.979 | 0.978 | 30.7 | **<0.001** | **<0.001** | 2.0 | 0.194 | 0.139 |
|  | Root starch |  | 0.3 | 0.593 | 0.518 | 9.5 | **0.015** | **<0.001** | 1.9 | 0.203 | 0.105 |
|  | *Micro-organisms* |  |  |  |  |  |  |  |  |  |  |
|  | AM fungi | mg_C_/m^2^_0-7cm_ | 0.8 | 0.391 | 0.200 | 0.1 | 0.732 | 0.607 | 0.3 | 0.581 | 0.412 |
|  | Sapro. fungi |  | 0.2 | 0.658 | 0.576 | 11.8 | **0.009** | **<0.001** | 0.1 | 0.727 | 0.672 |
|  | G(-) bacteria |  | 0.0 | 0.989 | 0.981 | 2.0 | 0.200 | ***0.052*** | 0.3 | 0.591 | 0.430 |
|  | G(+) bacteria |  | 0.0 | 0.935 | 0.910 | 6.3 | **0.036** | **<0.001** | 0.1 | 0.818 | 0.767 |
|  | Actinobacteria |  | 0.0 | 0.878 | 0.819 | 0.1 | 0.742 | 0.621 | 0.1 | 0.754 | 0.637 |
|  | (A+S)-F:B | - | 2.3 | 0.166 | **0.041** | 0.0 | 0.916 | 0.881 | 0.1 | 0.822 | 0.739 |
| **Resilience** | *General* |  |  |  |  |  |  |  |  |  |  |
|  | SWC | mass-% | 1.6 | 0.237 | 0.108 | 1.1 | 0.328 | 0.200 | 1.5 | 0.252 | 0.122 |
|  | Fine roots | g/m^2^ | 3.1 | 0.115 | 0.077 | 25.0 | **0.001** | **<0.001** | 9.6 | **0.015** | **0.005** |
|  | Total ^13^C uptake | mg/m^2^ | 3.0 | 0.123 | **0.030** | 1.1 | 0.329 | 0.196 | 1.6 | 0.248 | 0.118 |
|  | Root resp. CO_2_ | nmol g_dm_^-1^ s^-1^ | 0.4 | 0.553 | 0.447 | 3.2 | 0.110 | **0.047** | 0.0 | 0.982 | 0.979 |
|  | Plant ^15^N uptake^c^ | mg/m^2^ | 6.8 | **0.031** | **0.005** | 8.9 | **0.017** | **<0.001** | 3.6 | 0.096 | **0.048** |
|  | *Carbohydrates* |  |  |  |  |  |  |  |  |  |  |
|  | Shoot sucrose | mg_C_/g_dm_ | 0.6 | 0.447 | 0.299 | 4.7 | 0.062 | **0.009** | 0.4 | 0.568 | 0.441 |
|  | Shoot fructan |  | 3.3 | 0.105 | **0.024** | 2.1 | 0.181 | 0.073 | 0.8 | 0.403 | 0.264 |
|  | Shoot starch |  | 0.3 | 0.576 | 0.496 | 7.3 | **0.027** | **<0.001** | 0.2 | 0.689 | 0.636 |
|  | Root sucrose |  | 0.0 | 0.910 | 0.885 | 4.3 | 0.072 | **0.011** | 3.0 | 0.120 | **0.031** |
|  | Root fructan |  | 2.1 | 0.188 | 0.121 | 20.2 | **0.002** | **<0.001** | 0.4 | 0.545 | 0.481 |
|  | Root starch |  | 0.1 | 0.752 | 0.674 | 3.6 | 0.095 | **0.031** | 2.9 | 0.130 | **0.046** |
|  | *Micro-organisms* |  |  |  |  |  |  |  |  |  |  |
|  | AM fungi | mg_C_/m^2^_0-7cm_ | 3.1 | 0.114 | **0.011** | 0.0 | 0.859 | 0.811 | 0.1 | 0.719 | 0.622 |
|  | Sapro. fungi |  | 0.1 | 0.743 | 0.675 | 4.4 | 0.069 | **<0.001** | 0.0 | 0.910 | 0.873 |
|  | G(-) bacteria |  | 0.5 | 0.521 | 0.344 | 0.1 | 0.778 | 0.676 | 0.0 | 0.998 | 0.996 |
|  | G(+) bacteria |  | 0.7 | 0.441 | 0.265 | 0.7 | 0.422 | 0.245 | 0.5 | 0.505 | 0.341 |
|  | Actinobacteria |  | 0.1 | 0.755 | 0.643 | 1.1 | 0.333 | 0.144 | 0.1 | 0.775 | 0.663 |
|  | (A+S)-F:B | - | 4.4 | 0.068 | **0.009** | 0.1 | 0.749 | 0.676 | 0.5 | 0.499 | 0.346 |

(A+S)-F:B, (arbuscular mycorrhiza + saprotrophic) fungi:bacteria ratio; G(-/+), Gram-negative/positive; resp., respired; Sapro., saprotrophic; SWC, soil water content.

^a^*F*-values and approximate *P*-values from ordinary ANOVA (function ‘aov’ from the R base package); and ^b^exact *P*-values from distribution-independent permutational ANOVA (function ‘aovp’ from the
R package ‘lmPerm’); bold values, *P_F,exact_* < 0.05 (significant); bold italic values, *P_F,exact_* < 0.06 (nearly significant).

^c^The ^15^N addition was only done on monoliths used for the resilience labelling, plant ^15^N uptake is the sum of shoot- and root-incorporated ^15^N.

**TABLE S3** Effects of drought and land use on ^13^C tracer dynamics in above- and belowground plant parts and their carbohydrate pools, and in different soil microbial groups; after the resistance labelling (at peak drought) and after the resilience labelling (at recovery phase).

| **Labelling** | **^13^C incorporation in:** | **Unit** | **D** | | | **LU** | | | **Time** | | | **D × LU** | | | **D × Time** | | | **LU × Time** | | | **D × LU × Time** | | |
| --- | --- | --- | --- | --- | --- | --- | --- | --- | --- | --- | --- | --- | --- | --- | --- | --- | --- | --- | --- | --- | --- | --- | --- |
|  |  |  | df | *χ^2^* | *P* | df | *χ^2^* | *P* | df | *χ^2^* | *P* | df | *χ^2^* | *P* | df | *χ^2^* | *P* | df | *χ^2^* | *P* | df | *χ^2^* | *P* |
| **Resistance** | *Bulk plant material* |  |  |  |  |  |  |  |  |  |  |  |  |  |  |  |  |  |  |  |  |  |  |
|  | Shoot | mg_13C_/m^2^ | 1 | 11.9 | **<0.001** | 1 | 0.5 | 0.483 | 5 | 61.1 | **<0.001** | 1 | 3.1 | 0.077 | 5 | 13.7 | **0.017** | 5 | 4.3 | 0.510 | 5 | 7.9 | 0.161 |
|  | Root |  | 1 | 5.3 | **0.022** | 1 | 9.4 | **0.002** | 5 | 48.6 | **<0.001** | 1 | 0.1 | 0.747 | 5 | 2.2 | 0.816 | 5 | 11.7 | **0.039** | 5 | 9.4 | 0.096 |
|  | Root:shoot ratio | %_13C_ | 1 | 0.3 | 0.562 | 1 | 5.1 | **0.025** | 5 | 100.3 | **<0.001** | 1 | 3.2 | 0.074 | 5 | 10.0 | 0.076 | 5 | 7.9 | 0.164 | 5 | 5.0 | 0.412 |
|  | *Carbohydrates* |  |  |  |  |  |  |  |  |  |  |  |  |  |  |  |  |  |  |  |  |  |  |
|  | Shoot sucrose | mg_13C_/m^2^ | 1 | 0.3 | 0.588 | 1 | 0.1 | 0.773 | 3 | 97.2 | **<0.001** | 1 | 1.3 | 0.260 | 3 | 3.0 | 0.397 | 3 | 4.3 | 0.235 | 3 | 6.5 | 0.091 |
|  | Shoot fructan |  | 1 | 19.9 | **<0.001** | 1 | 0.2 | 0.651 | 3 | 11.5 | **0.009** | 1 | 0.0 | 0.920 | 3 | 1.6 | 0.663 | 3 | 0.9 | 0.830 | 3 | 1.2 | 0.752 |
|  | Shoot starch |  | 1 | 8.7 | **0.003** | 1 | 1.0 | 0.321 | 3 | 36.9 | **<0.001** | 1 | 5.7 | **0.017** | 3 | 6.4 | 0.095 | 3 | 0.7 | 0.864 | 3 | 2.6 | 0.454 |
|  | Root sucrose |  | 1 | 0.6 | 0.435 | 1 | 7.8 | **0.005** | 3 | 15.8 | **0.001** | 1 | 1.0 | 0.323 | 3 | 8.1 | **0.045** | 3 | 16.3 | **0.001** | 3 | 3.3 | 0.353 |
|  | Root fructan |  | 1 | 4.2 | **0.039** | 1 | 1.8 | 0.180 | 3 | 62.0 | **<0.001** | 1 | 4.1 | **0.043** | 3 | 7.7 | ***0.052*** | 3 | 5.6 | 0.131 | 3 | 3.5 | 0.317 |
|  | Root starch |  | 1 | 3.0 | 0.081 | 1 | 6.8 | **0.009** | 3 | 42.8 | **<0.001** | 1 | 2.1 | 0.145 | 3 | 4.9 | 0.177 | 3 | 3.0 | 0.394 | 3 | 7.1 | 0.067 |
|  | *Micro-organisms* |  |  |  |  |  |  |  |  |  |  |  |  |  |  |  |  |  |  |  |  |  |  |
|  | AM fungi | µg_13C_/m^2^ | 1 | 15.6 | **<0.001** | 1 | 1.7 | 0.194 | 1 | 0.7 | 0.389 | 1 | 2.8 | 0.097 | 1 | 2.6 | 0.107 | 1 | 1.5 | 0.223 | 1 | 0.6 | 0.449 |
|  | Saprotrophic fungi |  | 1 | 19.7 | **<0.001** | 1 | 0.1 | 0.770 | 1 | 5.0 | **0.026** | 1 | 3.3 | 0.068 | 1 | 0.1 | 0.752 | 1 | 4.1 | **0.043** | 1 | 2.0 | 0.159 |
|  | Gram(-) bacteria |  | 1 | 14.3 | **<0.001** | 1 | 0.4 | 0.512 | 1 | 0.2 | 0.629 | 1 | 4.7 | **0.030** | 1 | 6.1 | **0.013** | 1 | 1.7 | 0.197 | 1 | 0.7 | 0.399 |
|  | Gram(+) bacteria |  | 1 | 1.7 | 0.196 | 1 | 9.7 | **0.002** | 1 | 4.2 | **0.041** | 1 | 0.4 | 0.541 | 1 | 4.0 | **0.046** | 1 | 0.3 | 0.576 | 1 | 0.4 | 0.542 |
|  | Actinobacteria |  | 1 | 2.0 | 0.161 | 1 | 7.3 | **0.007** | 1 | 4.0 | **0.046** | 1 | 0.1 | 0.814 | 1 | 0.8 | 0.359 | 1 | 1.6 | 0.213 | 1 | 0.1 | 0.784 |
| **Resilience** | *Bulk plant material* |  |  |  |  |  |  |  |  |  |  |  |  |  |  |  |  |  |  |  |  |  |  |
|  | Bulk shoot | mg_13C_/m^2^ | 1 | 1.1 | 0.303 | 1 | 1.6 | 0.200 | 5 | 98.8 | **<0.001** | 1 | 0.8 | 0.381 | 5 | 7.5 | 0.189 | 5 | 14.4 | **0.013** | 5 | 18.7 | **0.002** |
|  | Bulk root |  | 1 | 0.2 | 0.628 | 1 | 4.9 | **0.027** | 5 | 30.7 | **<0.001** | 1 | 0.3 | 0.586 | 5 | 3.5 | 0.617 | 5 | 5.9 | 0.319 | 5 | 3.4 | 0.644 |
|  | Root:shoot ratio | %_13C_ | 1 | 0.2 | 0.683 | 1 | 4.1 | **0.042** | 5 | 103.1 | **<0.001** | 1 | 1.2 | 0.268 | 5 | 5.9 | 0.315 | 5 | 6.3 | 0.278 | 5 | 7.0 | 0.219 |
|  | *Carbohydrates* |  |  |  |  |  |  |  |  |  |  |  |  |  |  |  |  |  |  |  |  |  |  |
|  | Shoot sucrose | mg_13C_/m^2^ | 1 | 0.4 | 0.531 | 1 | 1.0 | 0.311 | 3 | 87.9 | **<0.001** | 1 | 0.1 | 0.822 | 3 | 4.9 | 0.177 | 3 | 9.0 | **0.030** | 3 | 2.8 | 0.431 |
|  | Shoot fructan |  | 1 | 0.1 | 0.749 | 1 | 0.1 | 0.779 | 3 | 3.4 | 0.329 | 1 | 0.0 | 0.991 | 3 | 4.7 | 0.194 | 3 | 4.9 | 0.176 | 3 | 1.1 | 0.772 |
|  | Shoot starch |  | 1 | 0.5 | 0.469 | 1 | 0.3 | 0.571 | 3 | 77.3 | **<0.001** | 1 | 0.0 | 0.996 | 3 | 1.8 | 0.609 | 3 | 0.2 | 0.972 | 3 | 4.6 | 0.203 |
|  | Root sucrose |  | 1 | 0.0 | 0.850 | 1 | 0.9 | 0.349 | 3 | 46.8 | **<0.001** | 1 | 6.8 | **0.009** | 3 | 1.5 | 0.694 | 3 | 2.0 | 0.573 | 3 | 0.4 | 0.930 |
|  | Root fructan |  | 1 | 0.9 | 0.342 | 1 | 0.8 | 0.385 | 3 | 44.1 | **<0.001** | 1 | 0.5 | 0.464 | 3 | 4.8 | 0.191 | 3 | 2.8 | 0.422 | 3 | 1.8 | 0.611 |
|  | Root starch |  | 1 | 0.0 | 0.865 | 1 | 1.6 | 0.201 | 3 | 37.4 | **<0.001** | 1 | 0.5 | 0.487 | 3 | 2.2 | 0.534 | 3 | 0.3 | 0.957 | 3 | 1.4 | 0.702 |
|  | *Micro-organisms* |  |  |  |  |  |  |  |  |  |  |  |  |  |  |  |  |  |  |  |  |  |  |
|  | AM fungi | µg_13C_/m^2^ | 1 | 3.6 | ***0.058*** | 1 | 0.4 | 0.549 | 1 | 0.8 | 0.376 | 1 | 1.2 | 0.280 | 1 | 0.2 | 0.648 | 1 | 2.0 | 0.156 | 1 | 0.3 | 0.608 |
|  | Saprotrophic fungi |  | 1 | 0.6 | 0.449 | 1 | 1.2 | 0.279 | 1 | 0.6 | 0.431 | 1 | 1.2 | 0.267 | 1 | 0.9 | 0.338 | 1 | 0.5 | 0.489 | 1 | 0.1 | 0.734 |
|  | Gram(-) bacteria |  | 1 | 1.3 | 0.256 | 1 | 0.0 | 0.889 | 1 | 0.8 | 0.386 | 1 | 5.5 | **0.020** | 1 | 0.2 | 0.671 | 1 | 0.9 | 0.349 | 1 | 0.1 | 0.816 |
|  | Gram(+) bacteria |  | 1 | 0.0 | 0.979 | 1 | 0.7 | 0.414 | 1 | 1.4 | 0.244 | 1 | 6.4 | **0.011** | 1 | 0.0 | 0.939 | 1 | 0.0 | 0.849 | 1 | 0.3 | 0.587 |
|  | Actinobacteria |  | 1 | 0.1 | 0.769 | 1 | 2.6 | 0.104 | 1 | 6.2 | **0.013** | 1 | 3.4 | 0.066 | 1 | 3.0 | 0.084 | 1 | 0.1 | 0.765 | 1 | 0.2 | 0.617 |

AM, arbuscular mycorrhiza; D, drought; LU, land use.

Statistics are based on linear mixed-effect models from the R package ‘lme4’; bold values, *P* < 0.05 (significant); bold italic values, *P* < 0.06 (nearly significant).

**TABLE S4** Mean residence time (MRT) of sucrose from shoots and roots of the abandoned grassland and the meadow, after the resistance and the recovery pulse labelling. MRT was calculated according to Hasibeder *et al.* (2015) and differences between MRT of drought and control treatments were tested using the exact Fisher-Pitman permutation test.

| **Labelling** | **C Pool** | **Land use** | **Treatment** | **Exponential fit^a^** | | | | **MRT (h)**  **mean** | **SE** | **P-value of**  **difference in MRT** |
| --- | --- | --- | --- | --- | --- | --- | --- | --- | --- | --- |
|  |  |  |  | Peak (h) | *n* | MRT (h) | SE |  |  |  |
| Resistance | Shoot sucrose | Abandoned | Control | 1.5 | 4 | 17 | 2 | 26 | 10 | 1.0 |
|  |  |  |  | 1.5 | 4 | 16 | 2 |  |  |  |
|  |  |  |  | 1.5 | 4 | 45 | 4 |  |  |  |
|  |  |  | Drought | 1.5 | 4 | 19 | 4 | 24 | 3 |  |
|  |  |  |  | 1.5 | 4 | 28 | 9 |  |  |  |
|  |  |  |  | 1.5 | 4 | 26 | 7 |  |  |  |
|  |  | Meadow | Control | 1.5 | 4 | 13 | 2 | 23 | 9 | 0.7 |
|  |  |  |  | 24 | 3 | 14 | 1 |  |  |  |
|  |  |  |  | 1.5 | 4 | 40 | 8 |  |  |  |
|  |  |  | Drought | 1.5 | 4 | 10 | 1 | 17 | 4 |  |
|  |  |  |  | 1.5 | 4 | 18 | 2 |  |  |  |
|  |  |  |  | 1.5 | 4 | 24 | 2 |  |  |  |
|  | Root sucrose | Abandoned | Control | 1.5 | 4 | 91 | 50 | 88 | 14 | 0.1 |
|  |  |  |  | 24 | 3 | 111 | 62 |  |  |  |
|  |  |  |  | 24 | 3 | 63 | 35 |  |  |  |
|  |  |  | Drought | 72 | 2 | ─ | ─ | 333 | 0 |  |
|  |  |  |  | 24 | 3 | 333 | 0 |  |  |  |
|  |  |  |  | 24 | 3 | 333 | 0 |  |  |  |
|  |  | Meadow | Control | 24 | 3 | 63 | 16 | 53 | 5 | 0.2 |
|  |  |  |  | 1.5 | 4 | 45 | 10 |  |  |  |
|  |  |  |  | 24 | 3 | 50 | 5 |  |  |  |
|  |  |  | Drought | 24 | 3 | 111 | 12 | 82 | 15 |  |
|  |  |  |  | 24 | 3 | 59 | 24 |  |  |  |
|  |  |  |  | 1.5 | 3 | 77 | 24 |  |  |  |
| Resilience | Shoot sucrose | Abandoned | Control | 1.5 | 4 | 14 | 6 | 11.2 | 1.4 | 0.7 |
|  |  |  |  | 1.5 | 4 | 11 | 4 |  |  |  |
|  |  |  |  | 1.5 | 4 | 9 | 0 |  |  |  |
|  |  |  | Drought | 1.5 | 4 | 8 | 1 | 10.6 | 1.3 |  |
|  |  |  |  | 1.5 | 4 | 11 | 4 |  |  |  |
|  |  |  |  | 1.5 | 4 | 13 | 0 |  |  |  |
|  |  | Meadow | Control | 1.5 | 4 | 14 | 2 | 14.1 | 0.5 | 0.1 |
|  |  |  |  | 1.5 | 4 | 14 | 6 |  |  |  |
|  |  |  |  | 1.5 | 4 | 15 | 1 |  |  |  |
|  |  |  | Drought | 1.5 | 4 | 12 | 0 | 11.1 | 0.3 |  |
|  |  |  |  | 1.5 | 4 | 11 | 1 |  |  |  |
|  |  |  |  | 1.5 | 4 | 11 | 2 |  |  |  |
|  | Root sucrose | Abandoned | Control | 72 | 2 | ─ | ─ | 39.3 | 10.7 | 0.3 |
|  |  |  |  | 1.5 | 4 | 50 | 10 |  |  |  |
|  |  |  |  | 24 | 3 | 29 | 2 |  |  |  |
|  |  |  | Drought | 1.5 | 4 | 50 | 3 | 49.4 | 2.1 |  |
|  |  |  |  | 1.5 | 4 | 53 | 28 |  |  |  |
|  |  |  |  | 24 | 3 | 45 | 2 |  |  |  |
|  |  | Meadow | Control | 24 | 3 | 24 | 14 | 48.7 | 21.2 | 0.7 |
|  |  |  |  | 1.5 | 4 | 31 | 7 |  |  |  |
|  |  |  |  | 1.5 | 4 | 91 | 33 |  |  |  |
|  |  |  | Drought | 1.5 | 4 | 34 | 2 | 34.7 | 8.8 |  |
|  |  |  |  | 1.5 | 4 | 20 | 6 |  |  |  |
|  |  |  |  | 24 | 3 | 50 | 0 |  |  |  |

^a^Equation *N = N_Peak_* e^-λ t^ fitted to *n* data points using nonlinear least squares regression, Peak refers to the time after labelling at which the ^13^C label was highest for each monolith,
MRT = λ^-1^.

**
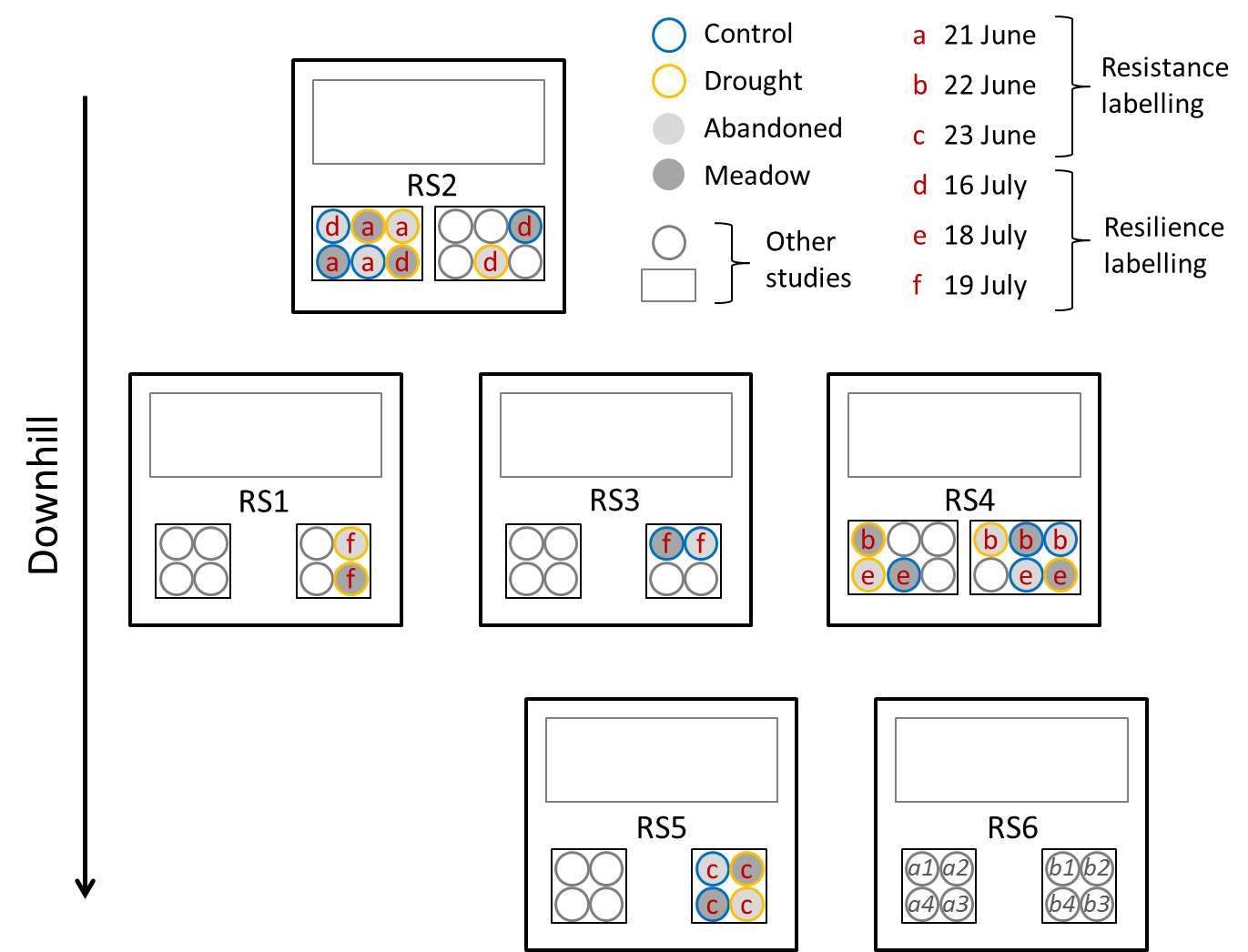
**

**FIGURE S1** Arrangement of monoliths on the experimental field in the Stubai valley on a spot with small inclination; the emphasised monoliths used for ^13^C pulse labelling in this study represent a subset from a bigger project (see also Ingrisch *et al.* 2017), which was set up in a randomized block design using six rain-out shelters (RS1-6) in total. Numbering of individual monoliths, as found in the data deposited in the Dryad repository (Karlowsky *et al.* 2017), was done as shown in RS6.

**
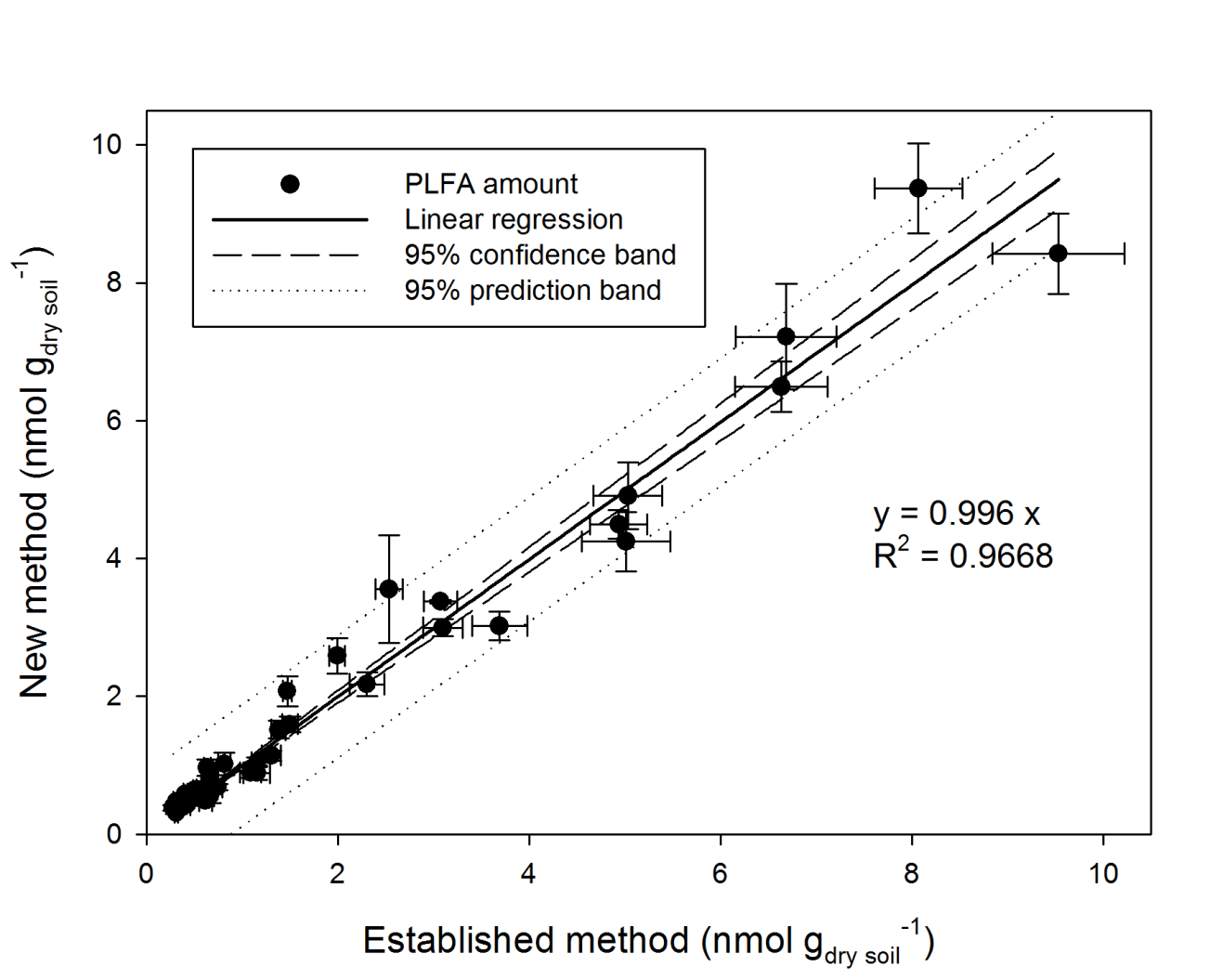
**

**FIGURE S2** Comparison of the extracted PLFA amounts between the new PLFA extraction method (pressurized extraction at 40 °C) and the established extraction method described by Kramer & Gleixner (2006); measured on the same soil sample from an arable field. Error bars show ± SD of n = 3 (new method) or n = 4 (established method) extractions.

**
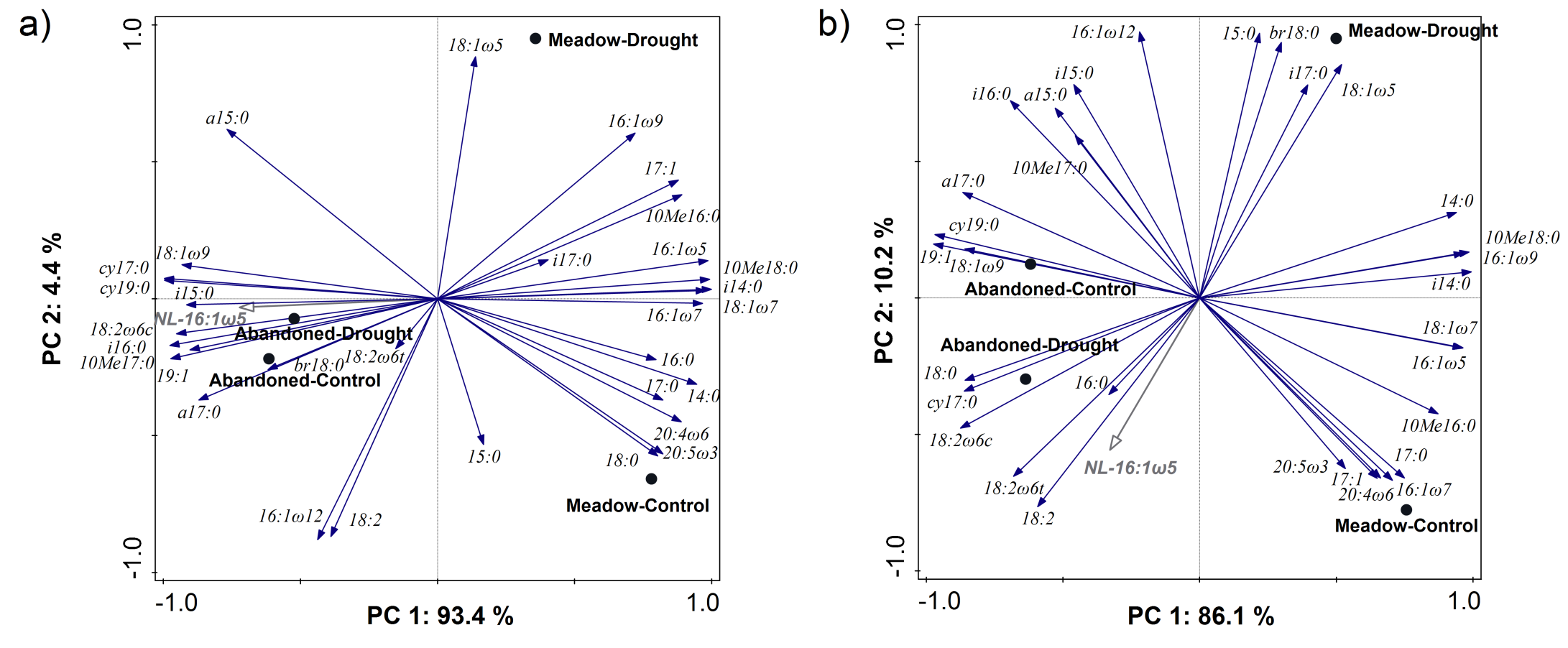
**

**FIGURE S3** Principal component analyses (PCAs) of PLFA abundances in mol-% a) at peak drought (resistance) and b) in the recovery phase (resilience) in control and drought monoliths of abandoned grassland and meadow. The content of the NLFA 16:1ω5 is added as supplementary variable. The PCAs were done using the Canoco 5 software (Microcomputer Power, Ithaca, USA) on log-transformed data (as recommended by the software).

**
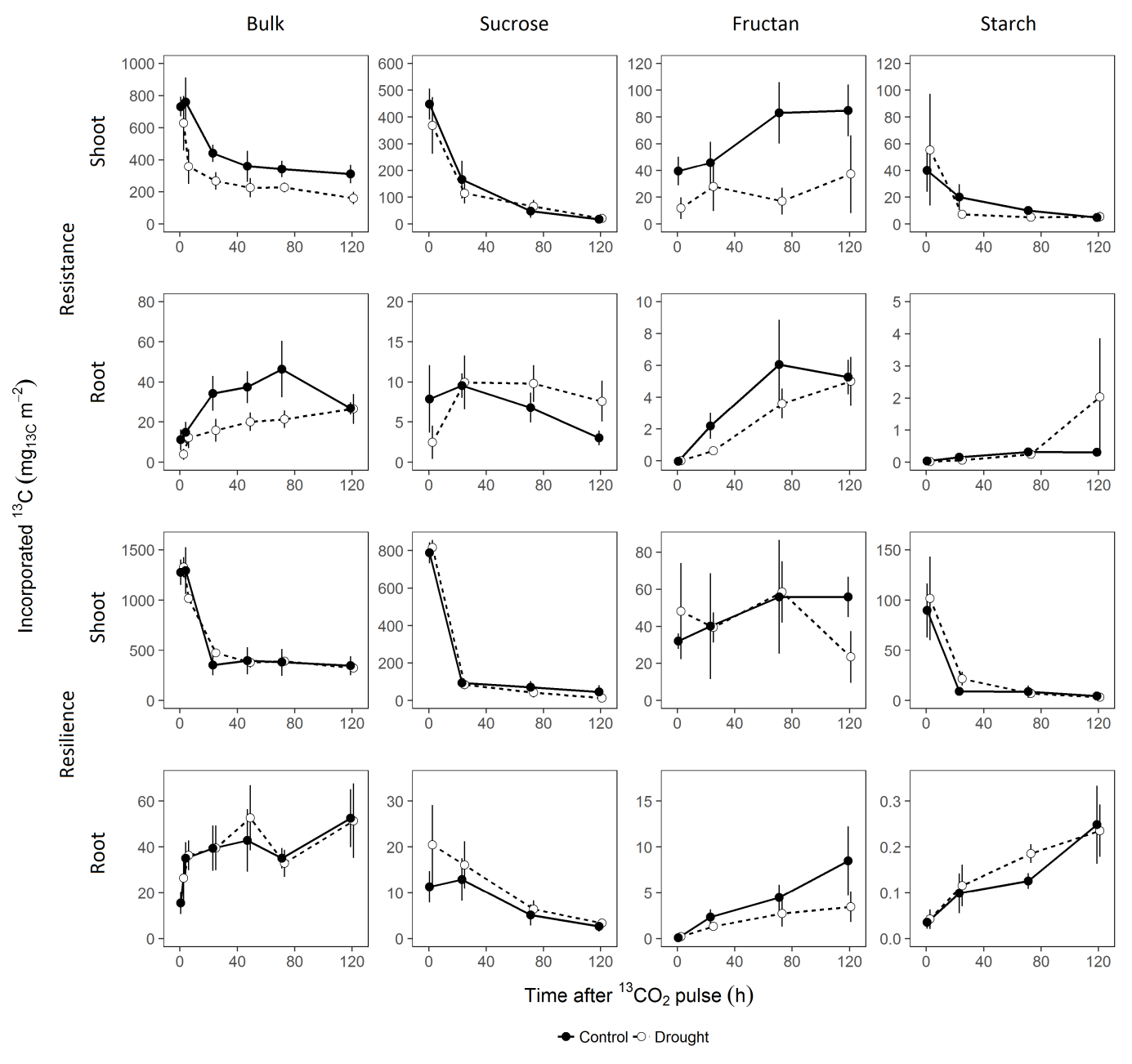
**

**FIGURE S4** Abandoned grassland: ^13^C tracer dynamics in bulk samples, sucrose, fructan and starch from shoots and roots of control (closed circles, solid line) and drought (open circles, dashed line) monoliths; after ^13^C pulse labelling during peak drought (Resistance, top) and after rewetting (Resilience, bottom). Error bars show ± SE (n = 3).

**
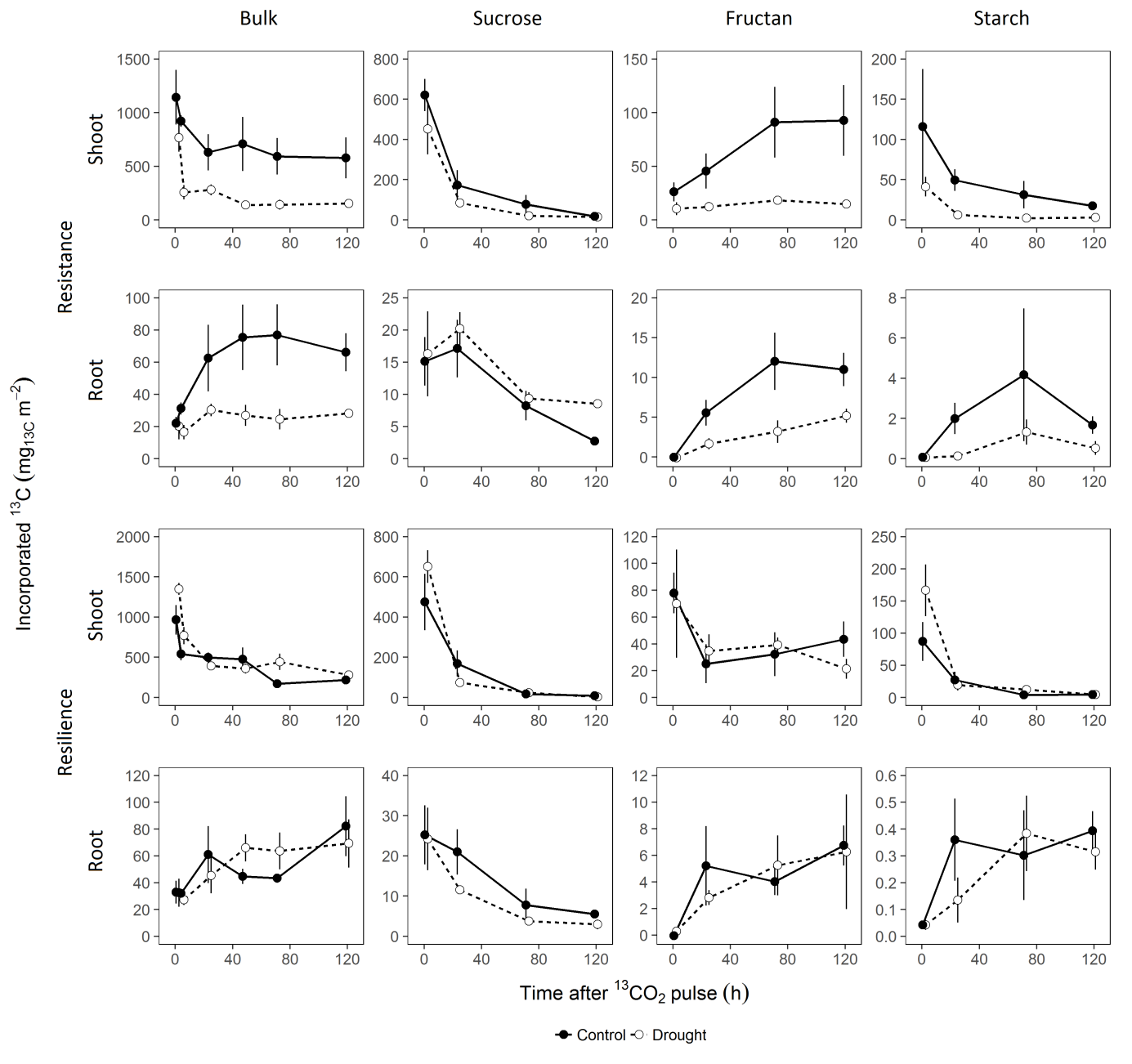
**

**FIGURE S5** Meadow: ^13^C tracer dynamics in bulk samples, sucrose, fructan and starch from shoots and roots of control (closed circles, solid line) and drought (open circles, dashed line) monoliths; after ^13^C pulse labelling during peak drought (Resistance, top) and during recovery (Resilience, bottom). Error bars show ± SE (n = 3).


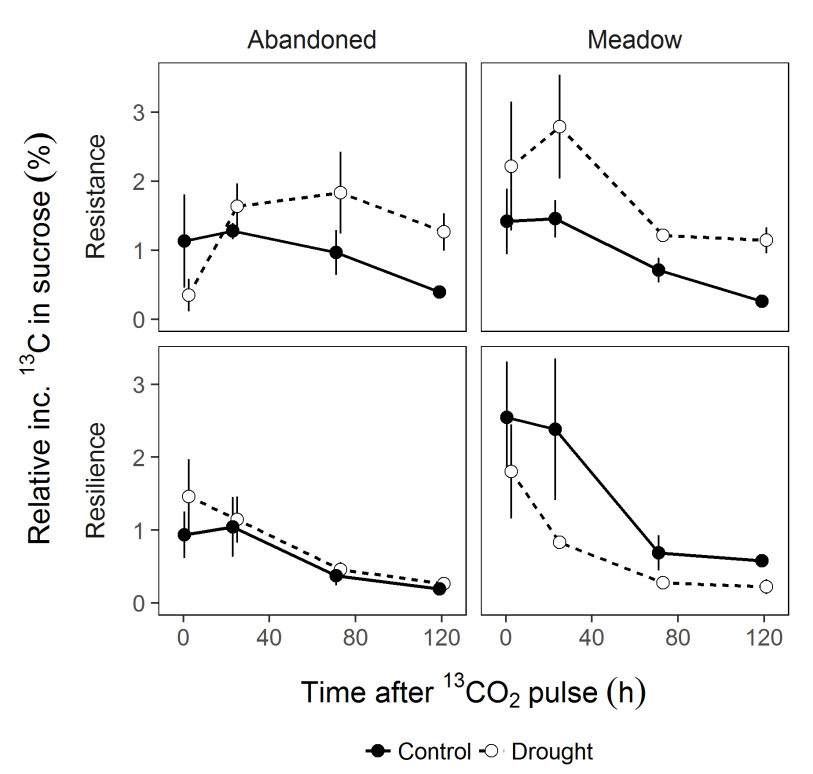


**FIGURE S6** Relative incorporated ^13^C (in % compared to the total ^13^C uptake in µg_13C_ m^-2^) in root sucrose of control (closed circles, solid line) and drought (open circles, dashed line) monoliths; from the abandoned grassland (left) and the meadow (right); after ^13^C pulse labelling during peak drought (Resistance, top) and during recovery (Resilience, bottom). Error bars show ± SE (n = 3)*.*

**
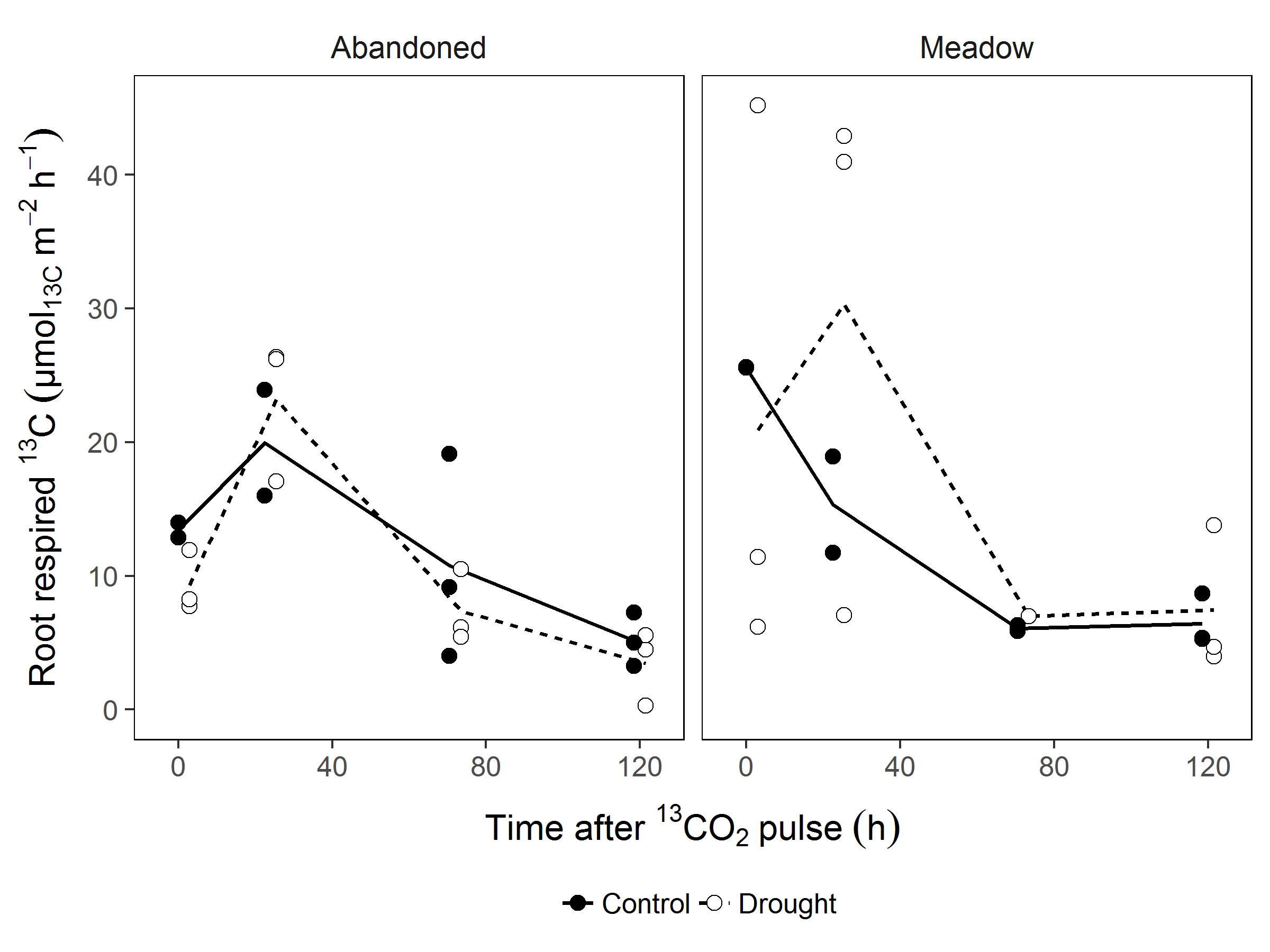
**

**FIGURE S7** ^13^C tracer dynamics of root respired CO_2_ at 15 °C from abandoned grassland (left) and meadow (right) after the resilience pulse labelling (2½ weeks after termination of drought). Depicted are single measurements points as circles and corresponding average values as lines of control (closed circles, solid line) and drought (open circles, dashed line) monoliths.

**
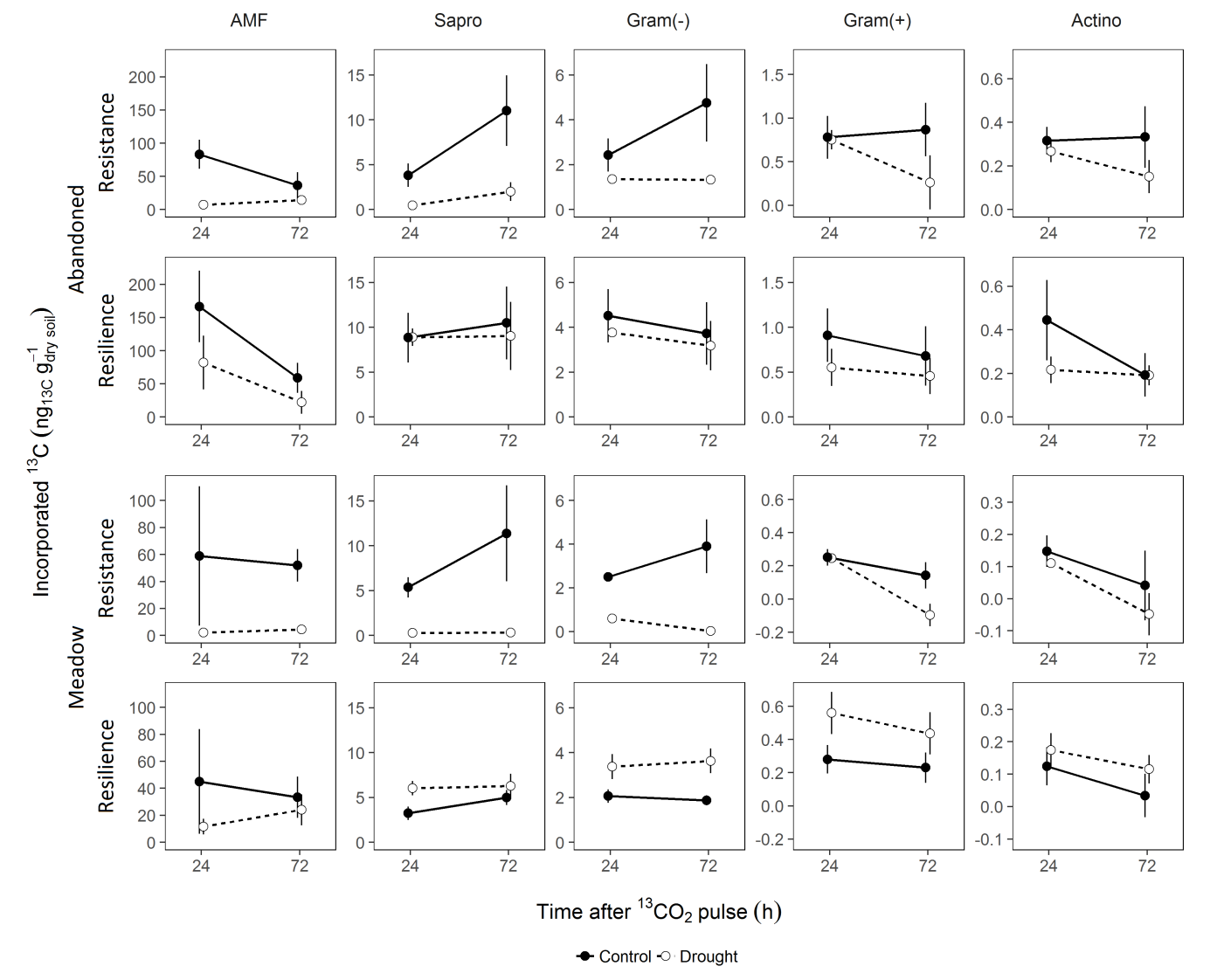
**

**FIGURE S8** ^13^C tracer dynamics in marker fatty acids for arbuscular mycorrhiza fungi (AMF), saprotrophic fungi (Sapro), Gram-negative bacteria (Gram(-)) and Gram-positive bacteria (Gram(+)), actinobacteria (Actino) of control (closed circles, solid line) and drought (open circles, dashed line) monoliths from abandoned grassland (top) and meadow (bottom); after ^13^C pulse labelling during peak drought (Resistance) and during recovery (Resilience). Error bars show ± SE (n = 3), except for AMF from meadow control monoliths 24 h after labelling (resistance and resilience) with ± SE (n = 2).

**References:**

Hasibeder, R., Fuchslueger, L., Richter, A. & Bahn, M. (2015) Summer drought alters carbon allocation to roots and root respiration in mountain grassland. *New Phytologist*, **205**, 1117–1127.

Ingrisch, J., Karlowsky, S., Anadon-Rosell, A., Hasibeder, R., König, A., Augusti, A., Gleixner, G. & Bahn, M. (2017) Land Use Alters the Drought Responses of Productivity and CO2 Fluxes in Mountain Grassland. *Ecosystems*.

Karlowsky, S., Augusti, A., Ingrisch, J., Hasibeder, R., Lange, M., Lavorel, S., Bahn, M. & Gleixner, G. (2017) Data from: Land use in mountain grasslands alters drought response and recovery of carbon allocation and plant-microbial interactions. *Dryad Digital Repository*, doi:10.5061/dryad.3s57p.

Kramer, C. & Gleixner, G. (2006) Variable use of plant- and soil-derived carbon by microorganisms in agricultural soils. *Soil Biology and Biochemistry*, **38**, 3267–3278.
